# Comparative Transcriptional Profiling of the Axolotl Limb Identifies a Tripartite Regeneration-Specific Gene Program

**DOI:** 10.1371/journal.pone.0061352

**Published:** 2013-05-01

**Authors:** Dunja Knapp, Herbert Schulz, Cynthia Alexander Rascon, Michael Volkmer, Juliane Scholz, Eugen Nacu, Mu Le, Sergey Novozhilov, Akira Tazaki, Stephanie Protze, Tina Jacob, Norbert Hubner, Bianca Habermann, Elly M. Tanaka

**Affiliations:** 1 Max Planck Institute of Molecular Cell Biology and Genetics, Dresden, Germany; 2 Max Delbrueck Center for Molecular Medicine, Berlin, Germany; 3 Max Planck Institute for Biology of Aging, Cologne, Germany; 4 Technical University Dresden, DFG Research Center for Regenerative Therapies Dresden, Dresden, Germany; Center for Regenerative Therapies Dresden, Germany

## Abstract

Understanding how the limb blastema is established after the initial wound healing response is an important aspect of regeneration research. Here we performed parallel expression profile time courses of healing lateral wounds versus amputated limbs in axolotl. This comparison between wound healing and regeneration allowed us to identify amputation-specific genes. By clustering the expression profiles of these samples, we could detect three distinguishable phases of gene expression – early wound healing followed by a transition-phase leading to establishment of the limb development program, which correspond to the three phases of limb regeneration that had been defined by morphological criteria. By focusing on the transition-phase, we identified 93 strictly amputation-associated genes many of which are implicated in oxidative-stress response, chromatin modification, epithelial development or limb development. We further classified the genes based on whether they were or were not significantly expressed in the developing limb bud. The specific localization of 53 selected candidates within the blastema was investigated by *in situ* hybridization. In summary, we identified a set of genes that are expressed specifically during regeneration and are therefore, likely candidates for the regulation of blastema formation.

## Introduction

In salamander, limb amputation initiates a wound-healing response followed by the emergence of a proliferative zone of cells, called the blastema, that consists of mesenchymal progenitor cells covered by an epithelium [1].

Injuries trigger a wound-healing response as the first step in regeneration, but simple wounding is not sufficient to launch a full regeneration response. A number of axolotl limb studies have indicated that limb wounds in the absence of full amputation are repaired imperfectly, as in mammals (for review see [2]). Moreover, critical size bone defects are not repaired in the axolotl limb, similar to mammals [3]–[5]. Therefore, the specific conditions related to amputating the limb are critical to the accumulation of mesenchymal blastema cells that will regenerate the limb. An important question is what are the molecular factors that determine the establishment of a blastema only after amputation, in contrast to other injuries.

In terms of a molecular perspective, a number of important studies have previously surveyed changes in gene or protein expression that occur during limb regeneration. Proteomic profiling at 1, 4 and 7 days after amputation and subtractive hybridization screen of the 4 day axolotl limb blastema compared to mature tissue have revealed a number of proteins and transcripts that are induced in a time course upon limb amputation [6], [7]. In these studies, the identified transcripts could have been associated with wound healing, amputation or both. Three additional studies using microarrays applied comparative strategies to delineate progress of normal limb regeneration versus conditions where regeneration fails. One study compared normal and denervated limbs at 5 and 14 days after amputation [8], [9]. Another study compared the regenerative versus laterally wounded epithelium at 7 days after injury, but the changes leading to the formation of mesenchymal blastema were not examined in this comparative approach [8], [9]. The most recent study used microarrays to profile normal and denervated limbs at 1, 3 and 7 days and compared that to a skin injury at the body flank [10].

While the events associated with wound healing are doubtlessly a critical part of initiating regeneration, our aim was to identify an amputation-specific gene set that underlies the transition from the adult to the blastema state, distilled apart from the wound healing gene network. It is likely that many changes occur in the first hours or days after limb injury, and a detailed time course particularly at the early time points may help to define the relative kinetics of gene expression changes required to define the early versus late genetic programs acting in this sequence.

We have identified a set of regeneration-associated genes in *Ambystoma mexicanum* (axolotl) by performing a high density expression profiling time course that compared healing of severe lateral wounds to regeneration of amputated limbs. We also measured expression in the developing limb bud, which was not described in previous studies. By comparing and bioinformatically clustering expression profiles of these samples, we observed a molecularly distinguishable tripartite program, which parallels the three phases of regeneration that were previously described based on morphological/cellular observations: early wound healing is followed by a transition-phase leading to establishment of the limb development program. By focusing on the transition-phase, we identified 93 regeneration-associated genes with annotated functions in oxidative-stress response, chromatin modification, epithelial development and limb development. In addition to the gene expression profiles identified in our microarray experiments, we provide an *in situ* hybridization database of the clearest regeneration-specific gene candidates that were identified in our screen. This dataset serves as a resource for gene products involved in converting cells to a regenerative phenotype.

## Results

### A screen to identify regeneration-specific transcripts in *Ambystoma mexicanum*

To carry out the gene expression profiling of regeneration, we produced a custom-designed 60-mer microarray based on an EST contig assembly of published EST-sequences from *Ambystoma mexicanum*, *Ambystoma tigrinum* plus unassembled salamander ESTs present in the NCBI database [11], [12] (Materials and Methods). In total this assembly consisted of 17452 non-overlapping contigs suitable for probe design. 9432 contigs were assigned a presumptive human homolog in the RefSeq protein database with a cut-off for homology at E =  10^−3^. In total we obtained 5792 different RefSeq identifiers.

For a subset of the contigs it was unclear which DNA strand is the coding strand, so for these contigs two strands were considered as separate targets and the probes were designed for both targets. Thus, in total we designed probes against 22753 different targets. In most cases (n = 20805) two non-overlapping probes were designed for each target, in a number of cases (n = 1926), a single probe, and more then two probes for 22 targets, giving a total of 43736 probes.

**Figure 1:**
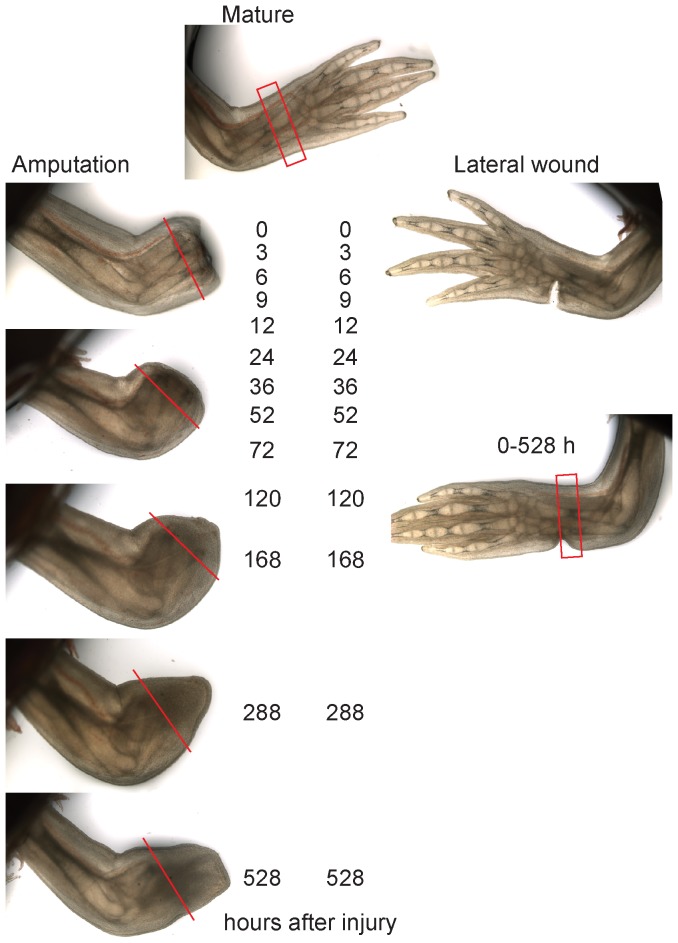
Comparative transcriptome profiling of regeneration versus lateral wounding in the axolotl limb. Live images of amputation and lateral wound limb samples spanning 0–528 hours (22 days) after injury. The red lines in the amputated series depict the plane approximately 2 mm behind the amputation plane. All tissue distal to the line was collected for the microarray sample. In the mature limb and lateral wound series the collected tissue is depicted by red rectangles.

We designed the expression profiling with the aim of identifying the earliest transcripts involved in blastema cell formation. We, therefore, compared forelimb lower arm regeneration versus deep lateral wound-healing samples in a high-density time course focusing particularly on early stages (0, 3, 6, 9, 12, 24, 36, 52, 72, 120, 168, 288 and 528 hours after injury). The latest time point represented the notch stage of regeneration, where the blastema had clearly started to re-differentiate into morphologically identifiable limb elements (Figure 1). To ensure that the amputated and lateral wound samples were as comparable as possible, the lateral wound samples were generated by slicing the forelimb through 50% of the cross-sectional diameter (Figure 1). Amputated and lateral wound samples were made as matched contralateral samples of four individual animals per time point and were separately pooled. In the amputated samples, the regenerated tissue along with tissue 2 mm behind the cut site was collected for processing. In the lateral wound samples a 2 mm thick limb segment just proximal to the injury was collected. In addition to the regenerating and wound-healing limb samples, we isolated RNA from developing axolotl limb buds pooled from different stages ranging from midbud (the earliest stage we could collect) to notch stage. Three independent time courses were performed to provide three biological replicates of the experiments.

The axolotl transcriptome is only partially sequenced. Although probes with an obvious cross-hybridizing potential were excluded from the array, it was important to assess the quality (potential cross-reactivity) of the probes. We compared the expression profiles between non-overlapping probe-pairs designed for the same target, focusing only on the set of 21493 probes that show statistically significant expression changes during the time course (ANOVA at 5% FDR). In this set there were 8151 probe-pairs and we calculated the Pearson's correlation coefficient (r) for each pair across all time points. For 88.3% of the probe-pairs r >0.8 and for 80.3% pairs r >0.9 (Figure S1A). Thus, for a majority of targets we find a good agreement in the expression measurement by independent probes strongly suggesting that they accurately reflect the corresponding RNA levels.

**Figure 2:**
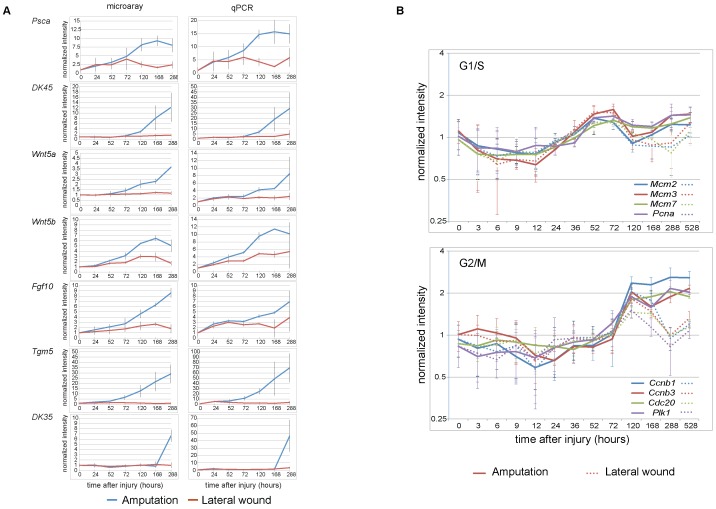
Validation of the gene expression profiling using microarray. A. Validation of gene expression changes by qPCR The expression profiles of seven representative genes- two with a strong signal from the microarray (*Psca and DK45 (RV_Am_asm_3322*)), two with medium signal (*Wnt5a and Wnt5b*) and three with a weak signal (*Fgf10, Tgm5 and DK35 (ET_Am_asm_6446*)) are shown. Time course of gene expression measured by microarray is shown on left, and qPCR on the right. The replicates of the microarray were normalized as described in the Methods section. qPCR data were normalized to the levels of *Rpl4* (Large ribosomal protein 4), which showed uniform expression levels in all microarray samples. In both, microarray and qPCR data, the normalized expression level at 0 hours is set to 1. The gene profiles obtained by microarray and by qPCR are remarkably similar although the dynamic range is 2–3 fold greater when measured by qPCR. All data points represent the mean of three biological replicates. Error bars show standard deviation. B. Validation of the time-course progression Cell cycle regulator expression reflects blastema formation in the regeneration time course. Top, G1/S-genes, *Mcm* and *Pcna* show a second peak at 288 hours in regeneration sample but not in the lateral wound sample. Bottom, G2/M-genes, *CyclinB, Plk, Cdc20*, remain highly expressed in the regeneration time course whereas they decline by 168 hours in the lateral wound time course. Each line depicts the trace of one representative probe for the gene averaged over three replicates with the error bars representing standard deviation. For each probe the median value of all measurements is set to 1. Solid lines: amputation time course, dotted line: lateral wound time course. See also Figure S1.

To validate the quantitative aspect of the gene expression data we performed qPCR analysis on seven representative targets; two targets with a strong signal in the microarray, two with medium signal and three with a weak signal. The median raw expression values of these seven genes in the microarray span three orders of magnitude. For all seven targets the microarray and qPCR profiles were remarkably similar, with qPCR generally showing about 2–3 fold larger difference between the baseline and the highest value per probe, as compared to the microarray (Figure 2A). This gave us confidence that a large percentage of the profiles would represent true changes in the expression profiles.

### A late regeneration-associated phase of cell cycle regulator expression

As an initial assessment of the time course dataset, we examined the profile of cell cycle regulators to determine if we could detect the onset of cell proliferation associated with injury and blastema formation. We charted the G1/S-phase transition associated *Mcm* factors and *Pcna*, and the G2/M-associated genes *Cyclins B1* and *B3*, *Cdc20 and Plk1* (Figure 2B). Consistent with their sequential roles in the cell cycle, the expression level of the G1/S-phase transcripts rose first, starting at 24 hours and rising to an initial peak at 72 hours. Transcripts associated with G2/M showed a peak at 120 h. This early response was observed in both amputation and lateral wound samples, indicating that the early onset of cell proliferation is associated with tissue injury. We conclude that the microarray profile can detect a cellular change associated with regeneration.

Interestingly, the regeneration samples specifically displayed a late phase of cell cycle regulator expression that corresponds to blastema cell formation/accumulation. A second wave of *Mcm* and *Pcna* expression rising noticeably by 288 hours is visible in the amputation samples and much less so in the lateral wound samples. Correspondingly, the G2/M-genes, *Ccnb1, Plk1* and *Cdc20* remain high post-120 hours in the amputated samples. A heat map of these and other cell cycle-related genes found in the experiment are provided in Figure S1B. These data show that the microarray expression profiling accurately reflects continued proliferation in the regenerating limb samples. Interestingly a similar “regeneration-specific” phase of cell division was observed in regenerating planaria [13].

### Clustering of expression profiles distinguishes three main phases: wound-healing, blastema establishment and reacquisition of the limb developmental gene program

**Figure 3:**
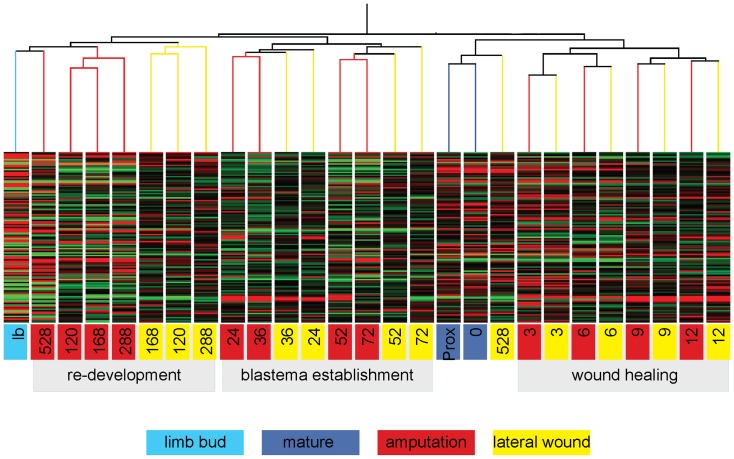
Hierarchical clustering of regeneration, lateral wound and limb bud samples identifies three main phases of limb regeneration. Samples were clustered using Pearson's correlation as similarity measure (sample key at bottom; numbers represent hours after injury, Prox =  mature sample from the upper arm at time 0 h, all other samples are from the lower arm). From 0–12 hours, at each time point, corresponding amputation and lateral wound samples cluster closest together. Starting at 24 hours, successive amputation samples are more similar to each other than to the corresponding lateral wound time points, indicating a divergence between regeneration and lateral wound gene programs. From 120 hours onward, the amputated samples are most similar to the developing limb bud, indicating that the limb development program has been re-established.

To gain an overview of the comparative time courses, we performed hierarchical clustering of the samples (using Pearson's correlation as the similarity measure). The data clustered into three main branches corresponding to early, middle and late time points. For the early time-points up to 12 hours, each corresponding lateral wound and amputation sample clustered closest together, suggesting a common wound-healing program (Figure 3). Starting from 24 hours up to 72 hours, the amputated samples of consecutive time points clustered more closely to each other compared to their cognate lateral wound samples, indicating that the amputation sample gene profile had started to diverge from the lateral wound samples. Finally, the 120 h to 528 h amputated samples clustered more closely with the developing limb bud than their corresponding lateral wound samples. This indicates that the 120 h–528 h limb blastema establishes a limb development-like gene program. Notably, the 528 h amputated sample most closely resembled the limb bud sample, while the 528 h lateral wound sample most closely resembled the un-amputated, mature limb sample, indicating that the 528 h lateral wound sample had approached adult tissue homeostasis. In summary, our analysis identified 24 hours as the time point at which the regeneration-specific gene program is first discernible from the wound-healing program.

### Identification and characterization of a regeneration-specific gene set

**Figure 4:**
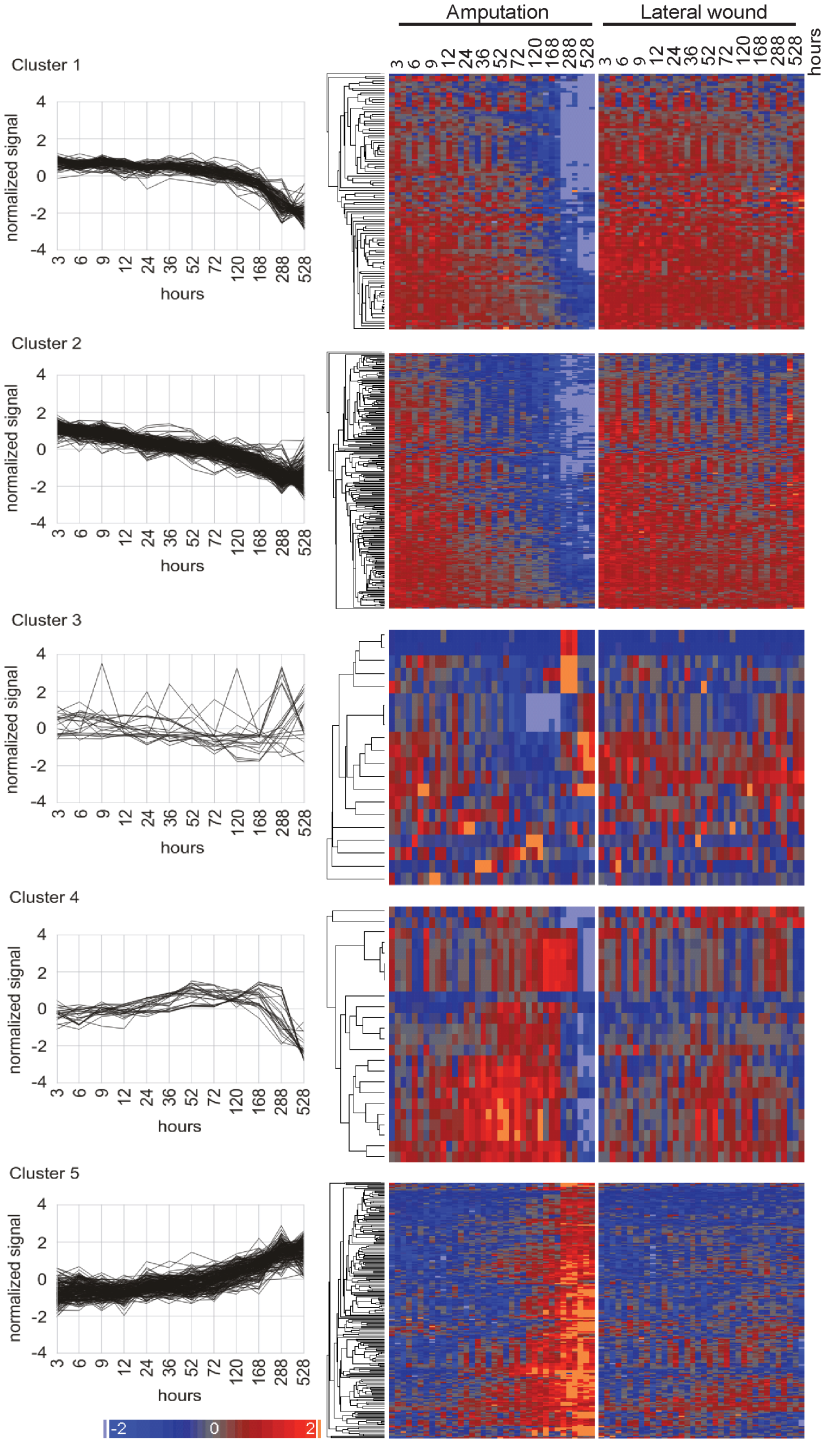
Two-way ANOVA analysis of parallel amputation and lateral wound time course yields regeneration-specific genes. Ribbon (left) and heat map (right) visualization of five signal clusters that emerged from 600 probes with amputation-specific regulation in limb. The heatmap shows log_2_ of standard scores of signal values (signal values normalized to a probe mean of zero and a standard deviation of one). Up-regulation is indicated by reds with increasing intensities, down-regulation by blues with increasing intensities. The genes in cluster 3, 4 and 5 were pursued.

We next sought to segregate a set of genes specifically involved in limb regeneration away from common wound-healing genes by comparing the amputation and lateral wound time courses. We performed two-way ANOVA analysis over the time course and between the amputation versus wound scenario resulting in 3645 probes with significant interaction effect (at 5% FDR). We further focused on those 600 probes that showed only insignificant expression changes over the wound time course (ANOVA p>0.05). Further sub-categorization of the amputation time course profiles of these 600 probes (median expression over replicates, see Experimental Procedures) resulted in five major clusters including two clusters with continuous (cluster 2) or sudden (cluster 1) down-regulation, one cluster with heterogeneous short transient patterns (cluster 3), one cluster with transient up-regulation (cluster 4) and one cluster with persistent up-regulation during regeneration (cluster 5) (Figure 4). The down-regulated clusters (cluster 1 and 2) were largely enriched in muscle-associated genes, presumably reflecting the degeneration of muscle at the amputation plane, and though likely to contain interesting genes, were not further considered. This left a list of 246 probes representing 194 different up-regulated targets (Table S1). Most of these targets (n = 189) were represented on our array by at least two probes. Pearson's correlation coefficient (r) between the expression values for the probe-pairs was greater than 0.8 for 142 targets (74%) confirming the reliability of the expression profiles obtained by the microarray.

From the list of 194 targets, 68 targets have no RefSeq assignment as they were below our cut-off for homology. The remaining 126 targets map to 93 different human RefSeq orthologs (Table S1).

**Figure 5:**
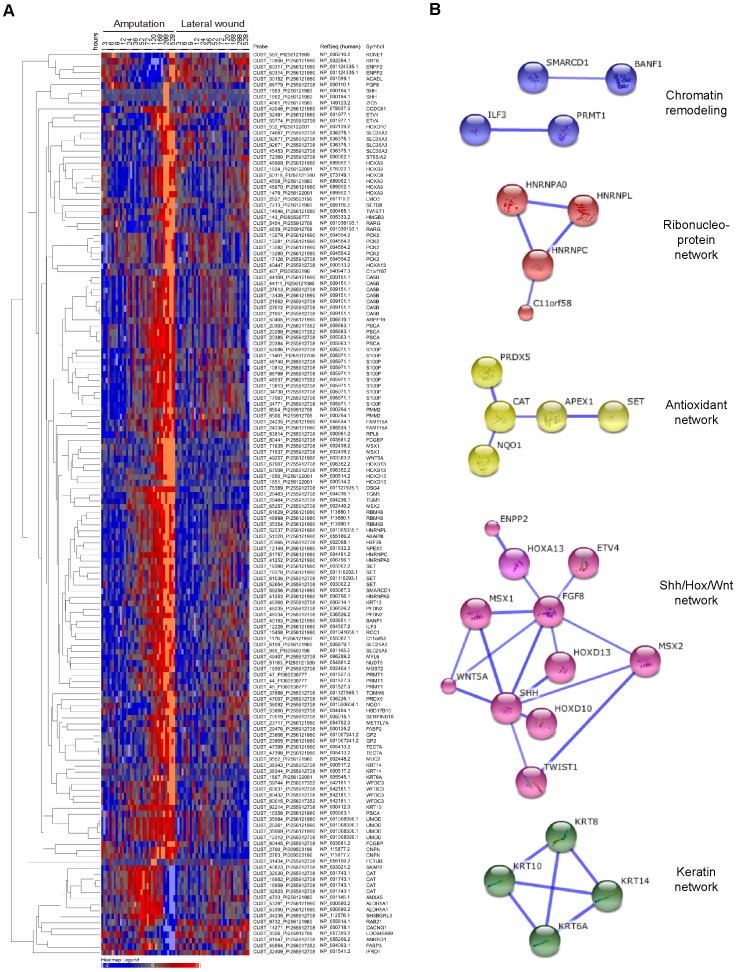
Similarity clustering and STRING analysis of the regeneration-specific genes identified by two-way ANOVA analysis. A. Heat map of 150 probes (with 93 different human homolog assignments) that are up-regulated in regenerating but not in lateral wound samples shows a diversity of expression kinetics. log_2_ values of standard scores of normalized signal are shown. Up-regulation is indicated by red, down-regulation by blue color. B. STRING analysis of the gene set showing only high confidence functional connections (confidence score ≥0.7). Five functional networks are identified: response to oxidative and cellular stress (yellow), ribonucleoprotein network (red), chromatin-remodeling (blue), epithelial cluster (green), development cluster (pink). Large balls represent gene families where a structure is available. Six identifiers were not recognized by STRING 9.0 interface and therefore 87 candidates were considered). See also Table S1, and Figure S5.

To gain an overview on the cellular processes represented in this set of 93 genes that are up-regulated in regenerating but not lateral wound tissue, we searched for evidence of enrichment in Gene Ontology (GO) categories using g:Profiler analysis tool [14]. Six RefSeq identifiers were rejected as ambiguous, so the enrichment was calculated using the remaining 85 entries. Based on GO terms there is a large enrichment in genes associated with limb morphogenesis, organogenesis, and epithelial development (Table 1). To gain a deeper insight into gene functions, a heat map of the gene list was used to visualize expression kinetics (Figure 5A), while potential physical and functional interactions were visualized using the STRING 9.0 interface. High-confidence interactions identified by STRING are shown in Figure 5B, while further potential interactions including low-confidence ones are shown in Figure S2 [15]. Several clear functional clusters can be seen, as color-coded in the STRING diagram.

**Table 1:** Gene ontology enrichment on the list of amputation-specific genes selected by two-way ANOVA analysis.

# Array	# List	P-value	GO term
1059	21	3.11E-09	tissue development
493	16	3.18E-10	epithelium development
389	10	7.02E-06	tissue morphogenesis
308	10	8.75E-07	morphogenesis of an epithelium
184	7	1.53E-05	morphogenesis of a branching structure
161	7	6.38E-06	morphogenesis of a branching epithelium
12	3	1.68E-05	branch elongation of an epithelium
2188	26	1.02E-06	organ development
260	10	1.85E-07	gland development
113	8	3.16E-08	gland morphogenesis
265	8	1.97E-05	epidermis development
704	13	1.01E-05	organ morphogenesis
185	8	1.40E-06	urogenital system development
223	8	5.61E-06	reproductive structure development
46	6	4.53E-08	prostate gland development
31	6	3.75E-09	prostate gland morphogenesis
30	6	3.04E-09	prostate gland epithelium morphogenesis
15	4	4.34E-07	branching involved in prostate gland morphogenesis
105	6	6.35E-06	male sex differentiation
98	6	4.26E-06	development of primary male sexual characteristics
240	9	9.75E-07	epithelial cell differentiation
33	5	2.94E-07	genitalia development
21	4	1.86E-06	male genitalia development
290	9	4.61E-06	regionalization
190	9	1.36E-07	anterior/posterior pattern formation
333	13	1.90E-09	skeletal system development
94	6	3.34E-06	embryonic skeletal system development
133	11	1.28E-11	appendage development
127	11	7.71E-12	appendage morphogenesis
133	11	1.28E-11	limb development
127	11	7.71E-12	limb morphogenesis
30	5	1.78E-07	hindlimb morphogenesis
29	5	1.49E-07	forelimb morphogenesis
811	14	9.50E-06	embryo development
442	12	4.41E-07	embryonic morphogenesis
111	11	1.73E-12	embryonic appendage morphogenesis
111	11	1.73E-12	embryonic limb morphogenesis
24	4	3.28E-06	embryonic forelimb morphogenesis
751	14	3.95E-06	sequence-specific DNA binding

Enrichment was calculated using g:Profiler on the list of 93 human RefSeq protein IDs corresponding to amputation-specific genes selected by two-way ANOVA analysis. 85 IDs from the list of 93 were recognized as unambiguous and were used to calculate the enrichment. Columns represent: #Array: total number of genes present on the array, that is associated to functional term, #List: number of genes in the input list that are associated to functional term, P-value: enrichment P-value, GO term: term name. The vertical alignment of the term name depicts its depth in the GO term hierarchy.

Here we highlight genes associated with four types of cellular function.

#### Cellular stress

**Figure 6:**
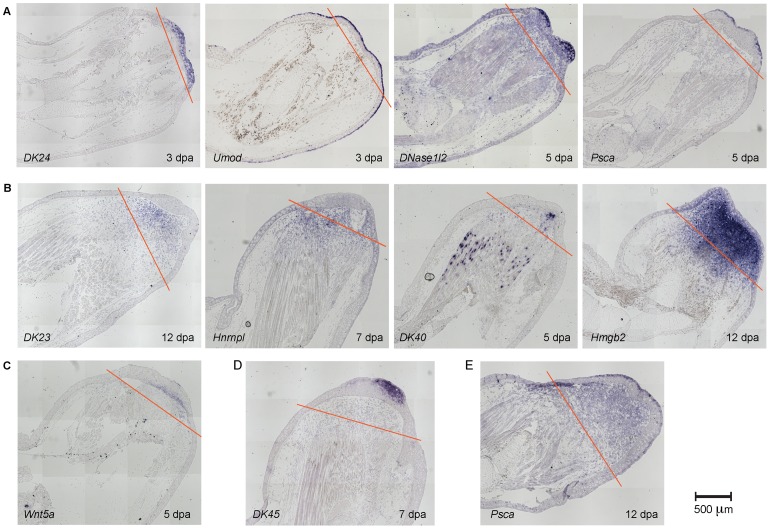
*In situ* hybridization on limb sections confirms a diversity of regeneration-specific expression patterns. A. Genes expressed in different layers of the wound epidermis. B. Genes expressed in the mesenchymal blastema or stump. C. Expression of *Wnt5a* in the basal epidermis of the amputated stump but not in the lateral injury. D. Expression of *DK45* in the limited region of the wound epidermis. E. In addition to wound epidermis, at 12 dpa, *Psca* is also expressed in the mesenchyme. Red lines depict the amputation plane. dpa =  days post amputation, dplw =  days post lateral wound. For comparison see the staining of lateral wound sections in Figure S3.

A number of genes (such as *Hnrnpl, Hnrnpa0, Hnrnpc, Prmt1, Ifrd1, Banf1, Ilf3, Mgst2, Nqo1, Prdx5, Slc25a5, Cat, Apex1*) are putatively linked with response to oxidative, metabolic or genotoxic stress, although the genes may be used for other cellular functions as well. Four genes from this group that were analysed by *in situ* hybridization (*Apex, Banf1, Hnrnpl, and Hnrnpc,*) all show expression in the mesenchyme of the blastema (Figure 6 and at http://est.age.mpg.de/cgi-bin/result.py). Our *in situ* hybridization expression data indicate that *Hnrnpl* is expressed in the mesenchymal blastema cells and in some cells behind the amputation plane (Figure 6) and unlike the other gene candidates shown in Figure 6 it is transiently expressed in the lateral wound (Figure S3). Although *Hnrnpl* up-regulation in the lateral injury time course was not statistically significant (ANOVA p>0.05), a mild up-regulation was also revealed by microarray. The various HNRNPs have been suggested to bind to 8-oxoguanine-containing RNA or transcripts such as p53 in response to oxidative or genotoxic stress [16], [17]. Furthermore, HNRNPL enhances translation of the VEGF riboswitch during hypoxia [18]. The protein arginine methyltransferase PRMT1 which methylates histone H4, mediates p53-dependent GADD45 induction upon DNA damage and also, PRMT1 mediated methylation alters the ability of ILF3 to bind DNA and promote transcriptional activation via nuclear hormone receptors [19]–[21].

Several aspects of the genes' expression suggest that they may function beyond generic metabolism and oxidative/genotoxic stress. The onset of expression for many of these genes started at 24 hours – a considerable lag to injury, and a time point long after the wound epithelium has covered the amputation surface. Furthermore, for most genes, expression remained high throughout the course of regeneration with the exception of *Cat* and *Ifrd1,* which showed transient up-regulation. Some members of this gene list were only up-regulated at late phases of regeneration (*Nqo1* and *Slc25a5* at 120 hours, and *Prdx5* at 288 hours). These features of the expression suggest a functional role in regeneration beyond generic metabolism.

#### Chromatin associated factors

A number of factors putatively involved in modulating chromatin were present in the gene set including *Prmt1, Smarcd1, Banf1, Set, Setd8, Hmgb3* and *Histone H3f3b. Smarcd1* (*Baf60a*) and *Prmt1* rise earliest at 24 hours. SMARCD1 constitutes one component of the SWI/SNF nucleosome-remodeling complex. In particular, SMARCD1 is part of an ES-cell specific form of the SWI/SNF complex that is involved in maintaining pluripotency [22]. Methylation of histones by PRMT1 plays an important role in gene regulation (for review see [23]). The *Set* gene whose expression increases at 36 hours is an oncogene that has been described to prevent acetylation of histone H4 [24]. *Banf1*, also increasing starting at 36 hours, is involved in recruiting chromatin to the inner nuclear membrane [25]. *H3f3b,* which rises at 52 hours, is a replacement histone that is able to exchange on chromatin in the absence of DNA replication. It has been described to be involved in chromatin remodeling in the germline, and modulation of gene expression at specific loci (for review see [26]). Finally, two factors rise at 120 hours, *Setd8*, a methyltransferase that targets histone H4K20 [27], and *Hmgb3* that is involved in regulating the balance between self-renewal and differentiation in hematopoietic stem cells [28]. Interestingly, *Mettl7a* encodes a putative methyltransferase of unknown function that rises at 120 hours. Further manual inspection of other chromatin modifiers such as *Smarcd4* and *Smarca5* showed a relatively strong induction in regeneration samples, with a mild induction in lateral wound (Figure S4). Apart from *Banf1,* which showed weak expression in the mesenchyme, we have no insight into the localization or identity of cells expressing genes from this group. Inspection of *Hmgb2*, which is not in our gene list, showed a modest regeneration-associated induction of gene expression in the microarray (Figure S4), but a strong induction in mesenchymal blastema cells by *in situ* hybridization (Figure 6).

#### Epithelial function and differentiation

Another significant class of genes is that associated with epithelial biology. An interesting aspect of the time course is the evident progression from a mucous epithelial to a cornified epithelial phenotype during the course of regeneration. A number of genes that express in the wound epithelium, increased expression by 24 hours, including *Krt10*, *Krt12, Psca*, *Tgm5*, and *Umod* (see Figure 6 for *Psca* and *Umod* expression). *Wnt5a* (Figure 6C), which is associated with appendage outgrowth [29], appeared at 52 hours, while *Fcgbp* and *Muc2* whose protein products are involved in forming mucous barriers [30], were expressed at 72 hours. Finally, transcripts associated with cornified epithelia including *Krt14*, *Dsg4*, and *Cnfn* increased at 120 hours with *Krt6* appearing at 528 hours. Genes expressed in the wound epidermis displayed a variety of different expression patterns in *in situ* stainings. While, *Krt14* was expressed very strongly in all the layers of the wound epidermis, *Gp2, Muc2, Pcsa* and *Umod* were restricted to the outermost layer, and Wnt5a was at the early time points found in the innermost layer of the epidermis (Figure 6). Several genes (*Dsg4, Cnfn, Fcgbp, Pcsa*, and *Wnt5a*) are expressed in the epidermis at early time (3–5 days), while at later times the expression is also observed in the mesenchyme of the blastema (Figure 6E).

#### Limb development module

As indicated by our initial hierarchical clustering, most of the transcripts associated with limb development rose late in the time course. Notably however, *Msx2* rose at 12 hours while *Aldh1a1,* which is involved in retinoid metabolism, was induced transiently from 36 to 168 hours. An interesting question is whether presence of retinoic acid at this stage plays a homologous function to that played during limb bud development. Subsequently, the transcripts encoding the extracellular factor *Wnt5a* and were induced at 72 hours. At 120 hours, the transcription factor *Twist1* and retinoic acid receptor gamma (*Rarg*), which was shown to play a role in proximal/distal identity [31] was induced, along with several *Hox* genes. Finally, at 288 hours, transcripts for *Shh* and *Fgf8,* which are involved in the outgrowth and patterning of developing limb buds appeared.

**Figure 7:**
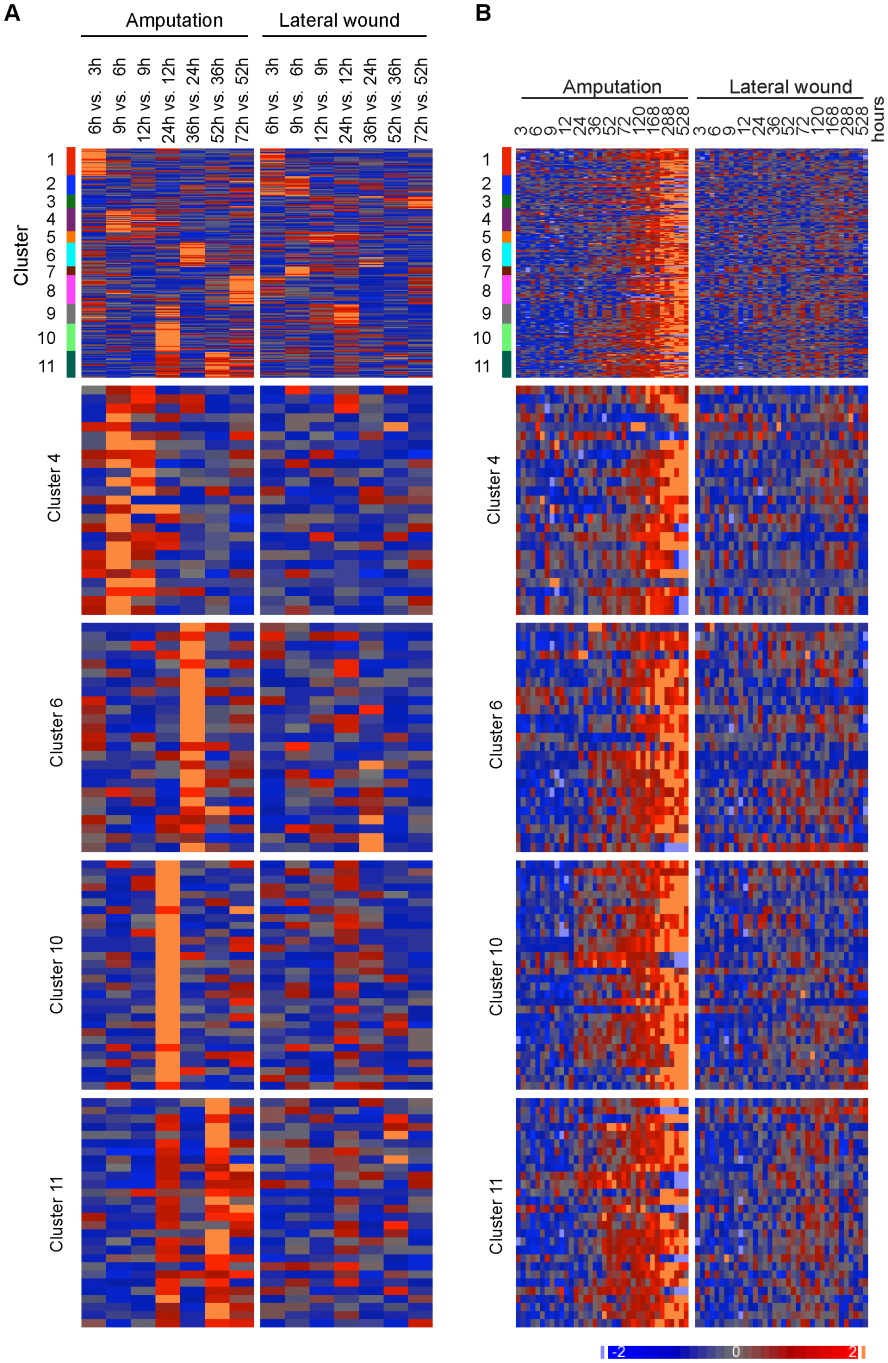
Selection of early regeneration-specific genes up-regulated during the blastema establishment-phase (3 and 72 hours after injury). A, B. Heat map visualization of all eleven p-value clusters that emerged from the 246 probes with up-regulation in limb amputation (top), and of four selected clusters with early up-regulation (4, 6, 10 and 11). A. Heat map of –log_10_ LSD p-values normalized to a mean of zero and a standard deviation of one. Values are calculated between consecutive time points from 3 to 72 hours after limb amputation and lateral wound. B. Heat map of log_2_ signal values normalized to a mean of zero and a standard deviation of one, shown for amputated limb and lateral wound between 3 and 528 hours after injury.

Since we were particularly interested in genes up-regulated during the blastema formation phase – up to 72 h after injury we further sub-categorized the 246 regeneration-associated probes using the Least Significant Difference (LSD) test p-values between consecutive time points in the time segment between 3 and 72 hours, for both the amputation and wound time course. Cluster 10 (Table S1) was selected for further investigation because of the early and relatively clear change from low to high expression between 12 and 24 hours in the amputation samples, and homogenous low expression in the lateral wound time course (Figure 7).

STRING network analysis of the genes in sub-cluster 10 showed only a weak direct connection between some members (*Etv4* and *St8sia2*). However, in relation to the other 246 probes some of the genes seem to be related to the *Shh–Hox* gene network (Figure S2).

### A supplementary regeneration-enriched gene set selected by pairwise comparison between amputation and lateral wound

**Figure 8:**
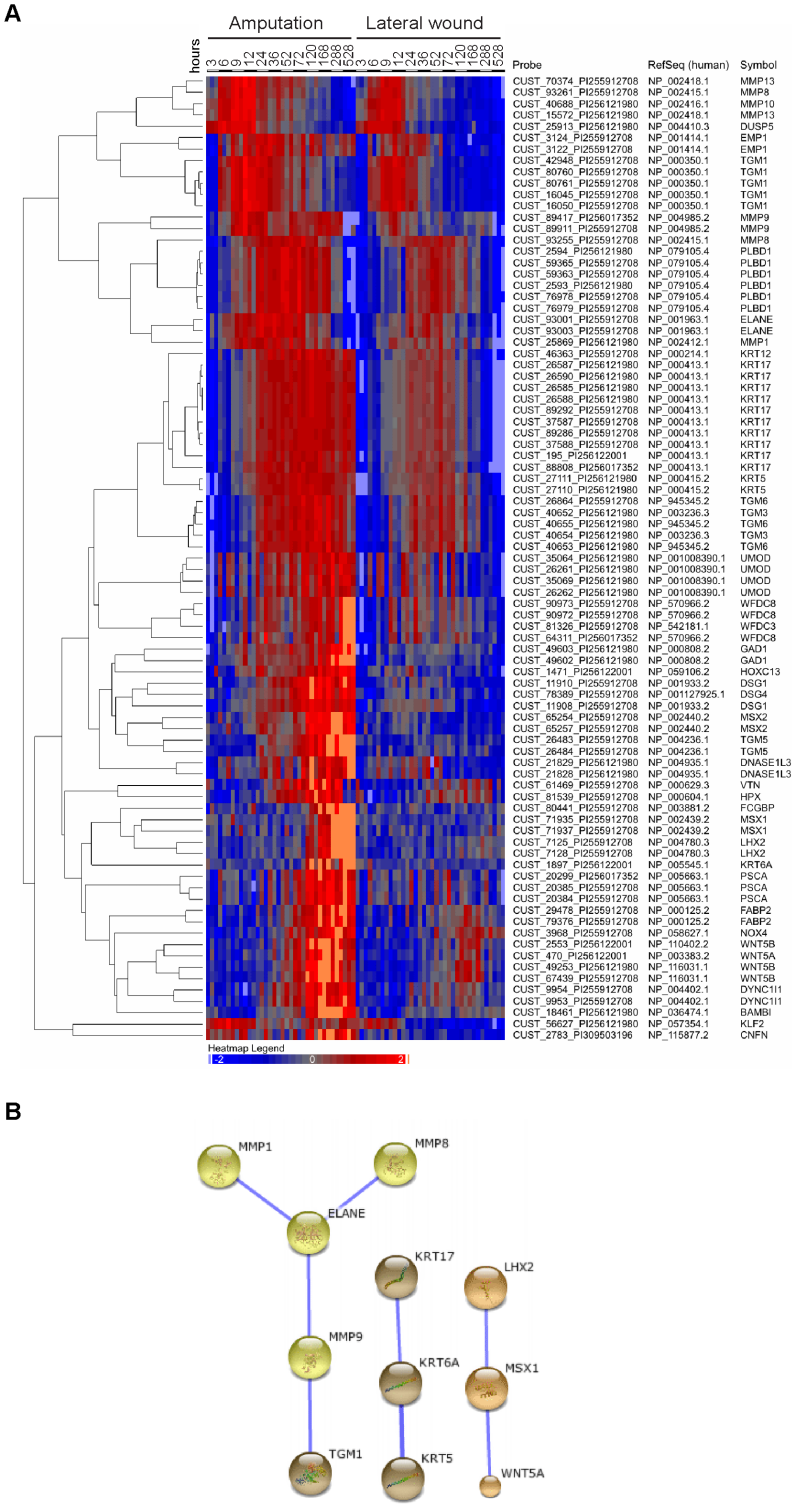
Similarity clustering and STRING analysis of the regeneration-enriched genes selected by pairwise comparison between amputation and lateral wound. A. Heat map of 85 probes (representing 44 genes with a assigned human Refseq homolog) up-regulated in regenerating but not or only weakly in the lateral wound samples shows a diversity of expression kinetics. log_2_ values of standard scores of normalized signal are shown. Up-regulation is indicated by reds with increasing intensities, down-regulation by blues with increasing intensities. B. STRING analysis of the gene set showing only high confidence functional connections (confidence score ≥0.7). ECM remodeling network including MMPs and elastase is shown in yellow, epithelial organization network in brown and the limb bud network including WNT5A, MSX1, LHX2 in orange color balls. Five identifiers were not recognized by STRING 9.0 interface and therefore 39 candidates were considered). See also Figure S5.

Histological comparison of the wounded versus amputated limbs has indicated that some aspects of the regeneration response may be initiated upon limb tissue injury, especially if nerve fibers have been severed [32], [33]. Considering the severity of our lateral wound injury protocol, we wanted to consider a gene set that may rise upon both amputation and lateral wounding but shows a lower induction and faster extinction in the lateral wound. To obtain this gene set we applied the following alternative criteria. We performed one-way ANOVA to identify genes that changed statistically significantly during the amputation time course (5% FDR) and obtained a list of 19017 probes, which comprises 43% of all the probes on our array. This is not unexpected considering that a multitude of processes is likely to be involved in regrowing a whole new limb, and highlights the need to apply comparative strategies when using gene expression profiling to identify genes critical for regeneration. In the second step, in order to identify genes that were significantly different between regenerating and the wound-healing tissue, we performed a pairwise comparison between amputation and lateral wound samples of corresponding time points (paired T-test, p<0.05). Considering that the cut area in the amputated sample is twice as large as in the lateral wound we applied a 2-fold difference cut-off. Finally, we selected only those probes that satisfied these criteria in at least three consecutive time points. From this analysis we obtained 583 probes, out of which 173 were up-regulated during the regeneration time course relative to the uninjured sample. These 173 probes corresponded to 114 contigs, including 52 contigs without assigned homology and 62 contigs with homology to 44 different entries in the human RefSeq database (Figure 8A, Table S2).

This dataset yielded some further candidates involved in oxidative stress such as *Nox4* and *Hpx* rising at 36 hours. Genes involved in epithelial cell organization include *Emp1*, *Tgm1,3,5,6*, *Krt5,6a*,*17* and *Dsg1*. Within the limb bud module, it identified *Msx1,2* and *Lhx2*. Several other limb bud associated genes were not selected by this approach because of their transient or only late up-regulation (ex. *Shh*, which is only up-regulated at 12 day blastema). In addition to the previous categories, this approach selected for an early up-regulated set of *Mmps 1,8,9,10,13* as well as *elastase*, which are involved in breaking down extracellular matrix at the injury site. The requirement of MMPs in limb regeneration has been tested via pharmacological agents [34]. The dataset also contained the transcription factor *Klf2* and *Bambi*, a protein involved in inhibiting BMP signaling [35]. Gene Ontologies enriched in this set of genes are related to two major themes: metabolic processes (in particular metabolism of the extracellular components) and tissue development (in particular epidermis development) (Table 2). STRING analysis identified three high-confidence networks: the elastin-metalloprotein network, the keratin network and the WNT5A, MSX1, LHX2 network (Figure 8B), and additional low confidence networks (Figure S5).

**Table 2:** Gene Ontology enrichment on the list of amputation-enriched genes selected by the pairwise comparison.

# Array	# List Listintersection	P-value	GO term
27	4	2.38E-07	peptide cross-linking
5637	29	6.50E-09	multicellular organismal process
61	5	1.39E-07	multicellular organismal metabolic process
27	5	1.98E-09	multicellular organismal catabolic process
4084	22	1.48E-06	developmental process
3471	22	7.65E-08	anatomical structure development
1059	18	3.47E-13	tissue development
493	8	5.12E-06	epithelium development
3708	21	1.37E-06	multicellular organismal development
3120	20	3.96E-07	system development
2188	20	8.64E-10	organ development
265	10	8.72E-11	epidermis development
50	5	5.04E-08	multicellular organismal macromolecule metabolic process
47	5	3.66E-08	collagen metabolic process
21	5	5.05E-10	collagen catabolic process
107	5	2.32E-06	epidermal cell differentiation
92	5	1.10E-06	keratinocyte differentiation
2	2	3.95E-06	external encapsulating structure organization
2	2	3.95E-06	cell envelope organization
442	7	2.52E-05	embryonic morphogenesis
388	8	8.64E-07	extracellular matrix
329	8	2.48E-07	proteinaceous extracellular matrix
2191	15	1.08E-05	extracellular region
106	5	2.22E-06	metalloendopeptidase activity
21	4	8.20E-08	transferase activity, transferring amino-acyl groups
9	4	1.76E-09	protein-glutamine gamma-glutamyltransferase activity
758	9	1.52E-05	calcium ion binding

Enrichment was calculated using g:Profiler on the list of 44 human RefSeq protein IDs corresponding to amputation-enriched genes selected by the pairwise comparison. 37 IDs from the list of 44 were recognized as unambiguous and were used to calculate the enrichment. Columns represent: #Array: total number of genes on the microarray that is associated to functional term, #List: number of genes in the input list that are associated to functional term, P-value: enrichment P-value, GO term: term name. The vertical alignment of the term name depicts its depth in the GO term hierarchy.

### Relationship of gene expression in the blastema versus the limb bud

Once the blastema has formed, the subsequent stages of regeneration resemble limb development [36]. Current and previous gene expression analyses support such observation [37]–[43], (Figure 3). However, the starting points for forming a blastema versus a limb bud are very different. While blastema progenitors, which are activated by injury, co-exist in the mature tissue with the cells that may not contribute to the blastema, limb bud progenitors are localized as discrete populations within main body axis and are induced at the appropriate time to contribute to the limb bud by intrinsic cell interactions. We, therefore, predicted that there would be genes in our dataset that are expressed in the blastema but not in the limb bud.

**Figure 9:**
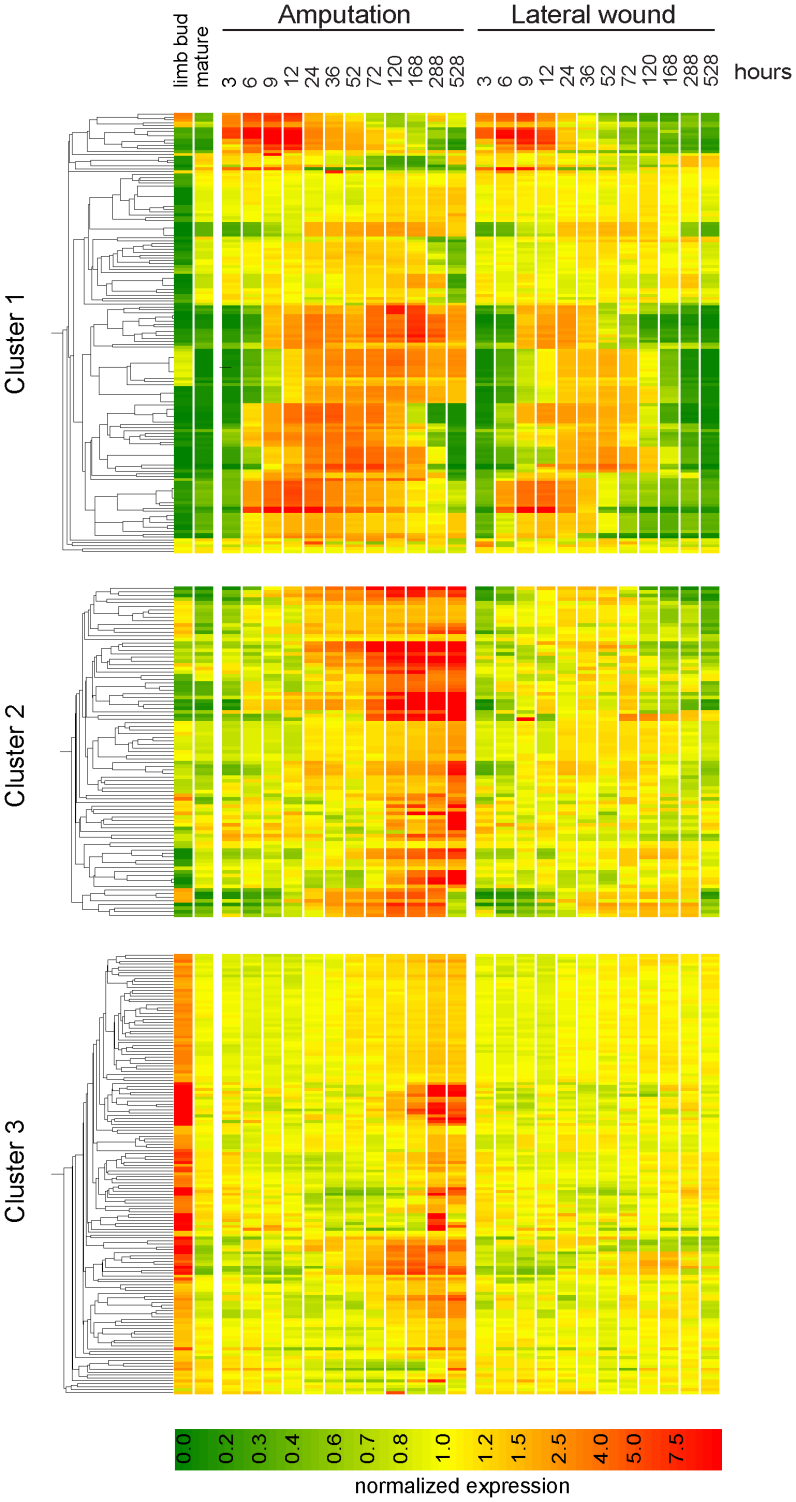
Heat map of 395 probes (all probes selected either by two-way ANOVA or by pairwise comparison) relating gene expression after injury with the expression in the limb bud. Three clusters were identified by K-means clustering. Gene trees are made on each of the clusters separately by using Pearson's correlation as similarity measure. Genes in clusters 1 (A) and 2 (B) are generally expressed at low levels in the limb bud, while genes in the cluster 3 were highly expressed in the limb bud. For identification of individual probes shown in the heat map see Figure S6.

Indeed, the amputation-specific and amputation-enriched datasets could be further sub-categorized into two classes – a group of genes (44%) that displayed robust expression in the limb bud, and another group (56%) with no/little detectable expression developing limb bud sample, pointing to a possible regeneration-specific function (Clusters 1 and 2 in Figure 9, and Figure S6A, S6B). The majority of the “limb-bud-high” genes were expressed in the “limb development” phase of the amputation time course, rising after 52–72 h and often peaking at 288 or 528 hours (Cluster 3 in Figure 9, Figure S6C). Not surprisingly, this gene set shows a strong enrichment of GO terms related to limb development and morphogenesis (Table S3). In contrast, genes with low expression in the limb bud were often up-regulated very early after injury (6–12 hours after injury). Many of these genes belong to the amputation- enriched dataset where limited expression is evident in the lateral injury (Cluster 1). These two “limb-bud-low” clusters show a strong enrichment in GO-terms related to epidermal development and differentiation, indicating differences between regenerating epidermis and the epidermis of the limb bud.

We propose three possible rationales underlying the “limb-bud-low” gene class. The most intriguing possibility is that these genes constitute an injury response that induces blastema cell formation, which would be consistent with their rapid activation after limb amputation. Alternatively these genes may be involved in differentiation into mature cell types such as larval skin, that are a natural feature of regenerating epidermis that are not yet found in the embryonic limb bud. Finally, it is possible that the limb bud mRNA sample contained a low representation of very early limb bud genes since it was physically only possible to collect limb buds equivalent to mid-bud to notch stage regenerates. Further investigation into the function of these genes will be required to resolve the role of these “limb-bud-low” amputation-specific genes.

### *In situ* hybridization database of blastema-associated genes

The regeneration blastema emerges from the mature tissue in the first days post amputation, and consists of two major compartments, the mesenchymal blastema that will give rise to skeleton, connective tissue, muscle, blood vessels and peripheral nerve, and the epithelium that plays an important signaling role during regeneration. To verify the regeneration-specific expression of the transcripts identified by this analysis, and to determine the spatial domains of their expression, we performed *in situ* hybridization of anti-sense RNA probes on sections of regenerating and laterally wounded samples at different time points after injury. We performed *in situ* hybridization for both, transcripts with a clear human ortholog, as well as un-annotated transcripts. We obtained interpretable expression patterns from 46 out of 53 transcripts examined. 40 out of 46 transcripts displayed regeneration-specific expression, validating our screening approach.

Figure 6 depicts representative expression patterns that we observed. We could classify the gene expression patterns into two broad categories: those that are expressed in the wound epithelium that directly covers the regeneration blastema (24 transcripts, Figure 6A), and those genes expressed in the mesenchymal blastema (16 transcripts, Figure 6B). The expression patterns in the epidermis revealed the complex structure of the epidermis, with molecularly distinct layers. For example *Gp2, Cnfn, axAg, Muc2, Umod, Psca, Dnase1l3* were all expressed from early time points in the outer layer of the wound epidermis. In contrast, *Dsg4*, *Krt17, Fcgbp, DK24, DK35, DK36, DK45* and *DK64* were expressed in all layers of the wound epidermis. Two genes were expressed in the innermost layer of epidermis: *Wnt5a* (Figure 6C) and *DK18*. Finally, *DK45* and *DK64* expression appeared to be localized to a limited area, usually the posterior wound epidermis (Figure 6D). *DK45, DK64* and *DK35* represent late onset genes whose expression becomes visible at 5 days after amputation (*DK45* and *64*) or at 7 days (*DK35*). A number of early wound epidermis genes also showed up-regulation in the blastema mesenchyme typically at later time points (12 transcripts such as *Pcsa (* Figure 6E *), Dnase1l2, Wnt5a*).

The mesenchymally expressed group of genes included: *Bambi, Gad1, Hmgb2, Hnrnpc, Hnrnpl, Tecta, Wnt5b, DK23, DK40* (Figure 6B). Robust expression in the mesenchymal blastema was most easily observable in later time points, such as 7 and 12 days. In most cases, the expression was uniformly distributed in the blastema. However, intriguingly, *DK40* marked a subset of cells in the mature tissue that were interspersed with muscle fibers. Whether these cells represent satellite cells or connective tissue, and whether the positive cells represent blastema cell precursors should be a topic of further investigation.

The expression profiling data, *in situ* hybridization images, EST contig assembly and BLAST server can be accessed in our Axologle database at: http://est.age.mpg.de/.

## Discussion

This work provides a detailed, parallel, time course of the regenerating limb blastema and of non-regenerative lateral wounds by microarray analysis. In addition, the present work compares the regeneration blastema and the limb bud. This analysis allowed us to identify a transition-phase between wound healing and re-establishment of a limb development program where a set of regeneration-specific genes is induced.

Based on morphological observations and expression analysis of selected genes in the injured limbs, it has been established that early wound healing lasting about 24 hours is comparable for both regenerating and lateral wounds [40], [44]. After that period the regeneration response begins to diverge from the injury/wound healing response such that only amputations but not lateral wounds lead to blastema formation. Once the blastema has formed, the subsequent steps resemble limb bud development. Thus, it was widely accepted that limb regeneration proceeds through three major phases: wound healing, blastema formation and re-development (reviewed in [45]). Our transcriptome-wide gene profiling is completely congruent with such a view, thus giving it further, independent support. Specific analysis of the cell-cycle associated gene set in this data revealed an initial rise in cell-cycle genes both in lateral wound and amputation time courses. However, a sustained expression of cell-cycle genes after 168 hours was only observed in the amputation time course. This continued expression of cell-cycle regulators in the amputated samples is consistent with the continued proliferation of the blastema for over one week prior to onset of overt differentiation between 288 and 528 hours. While no previous study specifically compared lateral wounds to amputated samples, ^3^H thymidine and mitotic indices have been extensively characterized in axolotl limb regeneration, and in non-regenerative samples where grafting of a mature skin piece onto the limb stump inhibited regeneration [46], [47]. A sustained rise in ^3^H thymidine labeling index, and a second rise of mitoses were observed in regenerating limb samples. In contrast, non-regenerative samples only showed an initial rise in ^3^H thymidine labeling followed by mitoses, but no sustained response. This published cellular data corresponds with the differences in cell-cycle gene expression changes we observed in amputation versus lateral wound time courses.

The purpose of our gene expression analysis was twofold. First, we wanted to expand the gene set that was previously implicated in regeneration by analyzing a comprehensive time course that is particularly dense at the early time points after injury. The second purpose was to classify the identified genes according to their putative involvement in the wound healing versus regeneration-specific events, by integrating the expression data resulting from limb amputation, a lateral limb injury and the developing limb bud.

Our data show that during the course of limb regeneration a large fraction of genes (>40%) undergo significant expression changes. 19017 probes (with 4087 different human protein annotations) detected statistically significant changes over the 22 days following amputation. Nearly a half of theses proteins (n = 2000) have not been implicated in limb regeneration based on previous expression analysis. Thus, the current analysis identified a substantial number of new genes whose expression is modulated upon limb amputation.

The proteomic analysis of blastema [6] identified 309 proteins with a significant fold change in amputated limbs relative to uninjured controls. About a half (55%) of these proteins were represented on our array, and our data show significant injury-induced changes at transcript level for 88% of them. Similarly, the majority of genes (83%, 95% and 89%) that were found up-regulated in three prior transcriptomic studies of limb regeneration ([9], [8] and [10]) and were represented on our array show statistically significant up-regulation in our regeneration time course, giving us confidence that our analysis was efficient in detecting changes in gene expression.

Because of the large number of genes involved in the process of regeneration, subtractive strategies are essential in order to start approaching the molecular aspects of blastema formation. Our strategy complements previous comparative approaches where nerve-dependent versus independent steps in regeneration, and regenerative versus non-regenerative epidermis were profiled using microarrays [8]–[10].

Comparing transcript levels between amputation and lateral injury revealed that the majority of gene expression changes that occur after amputation are not regeneration-specific. Only 6% of the genes that changed during limb regeneration showed preferential up-regulation during limb regeneration as compared to wound healing – 1183 probes detected amputation-specific/enriched changes compared to 19017 probes that detected statistically significant changes during the time course relative to the uninjured sample. Similar proportion of amputation-enriched genes may be expected in “regeneration” gene sets that were previously identified through the comparison of uninjured limb and the regenerating limb [6], [7]. Consistent with this expectation, only two genes (*Hnrnpl and Krt12*) in the proteomic set are amputation-specific/enriched according to our data.

We compared the amputation-specific/enriched set identified by the current study with three previous studies where comparative strategies were applied (Table S4). Two studies investigated the role of peripheral nerves in regeneration and identified genes that were up-regulation in the amputated limb in a nerve-dependent fashion. Interestingly, we found that only 12 out of 95 [8] and 2 out of 22 [10] nerve-dependent genes that were represented on our array qualify as amputation-specific/enriched. These results conform with the idea that nerve injury and large tissue removal create two initially separate inputs into regenerative response [33]. In addition, Monaghan et al. identified 377 “limb-specific ”annotated probe sets that were up-regulated in the amputated versus uninjured limb as well as in in amputated limb versus skin injury on the body flank [10]. 203 of these “limb-specific” genes were represented on our array and 26 of them were identified as amputation-specific/enriched. Conversely, from 112 annotated amputation-specific/enriched genes identified by current study, 56 also change the expression after amputation according to [10], and 26 are considered “limb-specific”.

A more substantial overlap exists between our amputation-specific/enriched gene set and the gene-set obtained by the study that compared regenerative epidermis of an amputated limb with a non-regenerative epidermis that covered a lateral injury at 7 days post injury. This approach was the most similar to ours in the design and also the same microarray platform was used. The identified regeneration-specific gene set included 58 candidates with putative human orthologs [9]. 16 of these genes (*Aldh1a1, Dnase1l3, Dsg1, Dync1i1, Gp2, Klf2, Krt5, Msx1, Msx2, Psca, S100P, Tecta, Tgm1, Tgm5, Umod, Wnt5a*) were shared between this gene-set and our 112 regeneration-specific/enriched candidates with putative human orthologs. The difference between the two gene lists likely reflects the difference in the tissue that was examined. The current study sampled whole blastemas, therefore it detected many genes that were up-regulated in the mesenchymal part of the blastema in addition to the epidermal genes detected by Campbell et al. On the other hand, we might have missed some of the subtle changes in the wound epidermis genes that were detected by Campbell, because a larger contribution of non-epidermal tissue would reduce sensitivity to detect such changes.

One part of our regeneration-specific gene set are genes putatively related to oxidative-stress. While it may have been expected that such a response would be common to generic wound healing, there is clearly a sustained, regeneration-specific expression of genes in this category. An intriguing question is whether the putative oxidative-response-associated gene set is involved in promoting or sustaining a proliferative, self-renewing state of blastema cells within an adult tissue context and counteracting senescence/aging. Stem cell self-renewal and maintenance is known to be highly sensitive to oxidative-stress (for review see [48]). An alternative possibility is that metabolites such as hydrogen peroxide play important signaling roles in regeneration, extrapolating on recent evidence that hydrogen peroxide is a key signaling molecule in wounded tissues [49]. The expression of some genes related to oxidative-stress only at the latest time points i.e. time of blastema differentiation, suggests that at least some of them play an important signaling role.

We also identified a set of genes associated with chromatin modification. Previous association of some of these factors with oxidative-stress, and with nuclear hormone signaling raise the intriguing possibility that these genes are involved in mediating putative extracellular signaling events that convert cells from the adult homeostatic condition toward a regeneration blastema cell type.

In terms of cellular reprogramming we examined whether those factors involved in reprogramming fibroblasts to pluripotency, *Oct4, Sox2, Nanog, Myc,* and *Klf2/4* were also significantly induced during limb regeneration. *Klf2/4* was highly induced in both limb regeneration and lateral wounding while *c-myc* was mildly induced at late time points after regeneration and wounding, but *Oct4*, *Sox2*, *Nanog* are not induced significantly and reproducibly in our analysis (Figure S4A). Previously, up-regulation of *Sox2* was reported in regenerating newt limb [50] but was not observed during limb regeneration in *Xenopus* [51]. The discrepancy with the results in newt could be due to differences in linearity between qPCR and microarray data. Furthermore, our data from different probes along the *Sox2* sequence are not consistent, indicating that further investigation is required.

Recently two piwi-like genes (*Pl1* and *Pl2*), which play crucial roles in the germ cells of mice and flies [52], were reported to be induced and essential for the blastema formation in the axolotl [53]. While *Pl1* was not represented on our array, we did not detect significant up-regulation of the *Pl2* during the limb regeneration time course.

Transcripts encoding for several extracellular signaling molecules that are potentially important for blastema formation and/or outgrowth are up-regulated during regeneration/wound healing according to our data. Wnt/β-catenin signaling plays an essential role during the early stages of limb regeneration [54], [55]. We find an early (3 h-24 h after injury) up-regulation of *Wnt10a* that was comparable between amputation and after lateral injury, and it appears to be a part of the stress response as its low expression in the limb bud suggests that *Wnt10a* is not a part of the limb development program in the axolotl. Other canonical *Wnts* (*Wnt1, 3* and *8*) showed no significant expression change during the time course. Only *Wnt5*s (particularly *Wnt5a*) were specifically enriched in the amputation-sample compared to the lateral wound. Previously it was shown that over-expression of *Wnt5a* early in axolotl limb regeneration inhibited the progression of limb regeneration and the maintenance of *Msx2* [56]. Over-expression at later stages had no effect confirming a role of *Wnt5* in the early steps of regeneration. Other studies in different animal systems point to different roles for *Wnt5* in regeneration so it is not yet clear whether there is a single unifying role for *Wnt5* in appendage outgrowth and regeneration [57], [58].

*Fgf*s have been implicated in regeneration as nerve-derived trophic factors necessary for maintenance of blastema proliferation [59], [60]. Also, *Fgf8* and *Fgf10* are essential apical ectoderm and mesenchymal components, respectively that are essential for outgrowth and patterning of the limb bud and regenerating limb. Both are expressed in *Xenopus* and axolotl limb blastemas but not in non-regenerative *Xenopus* limb [39], [61]. Our analysis shows a bimodal up-regulation of *Fgf8* – a weak early up-regulation that is comparable between amputation and lateral-injury samples, and a late amputation-specific peak at 12 days after amputation. On the other hand, *Fgf10* showed a steady up-regulation starting from 24 hours after injury, which is more pronounced in the amputated sample (Figure 2). Curiously, based on three independent probes we find no evidence of amputation-induced up-regulation of *Fgf20*, which has been identified as a key regulator necessary for blastema formation in the zebrafish tail regeneration [62]. It is unclear if the absence of *Fgf20* up-regulation in our experiments is due to detection sensitivity related issues or it reflects the actual differences between the two species/tissues.

*Bmp*s have been implicated in regeneration in *Xenopus*, axolotl and mouse limbs [63]–[66]. *Bmp2* and *Bmp7* were represented on our microarray. While *Bmp7* levels did not show any significant change during the time course, *Bmp2* showed two waves of expression in our experiment; an early up-regulation at 3–12 hours, which is comparable between amputated and lateral injury samples, is followed by a second wave of expression at 5–12 days, which is more pronounced in the amputated samples. This bimodal expression would be consistent with its proposed roles; first in progenitor cell proliferation and later in cartilage differentiation [67].

An interesting aspect of the epithelia associated genes was the progression from a mucous gene profile at early time points, to a cornified epithelial phenotype in the later time points. Since the wound epithelium is a crucial structure supporting limb regeneration, it will be important to determine if this mucous phenotype is required for productive limb regeneration. The requirement for Anterior Gradient, a protein associated with secretory glands during limb regeneration in *Notophthalmus viridescens*, points to the possible functional importance of a mucus epithelium in supporting regenerative outgrowths [68], [69]. A number of epidermis-expressed genes identified in this study as regeneration enriched (*Ca5b, Dnase1l3, Psca, Tgm5, Umod, Wnt5a*) overlap with genes identified by others including nerve dependent transcripts [8], [9], Table S4. Our *in situ* data confirms that many epithelia expressed genes are nerve dependent.

It has long been speculated that there are parallels between cancer and blastema formation. Although we find many oncogenes that are up-regulated during regeneration, most of them are subtracted from our regeneration-specific dataset because they are induced both by amputation and by wounding in axolotl. It is clear that wound-healing induced genes could be important to initiate some aspects of a regeneration response that are transient upon wounding, but that progress after limb amputation. The comparison here was made to identify regeneration-specific genes that arise early after wound healing. Further analysis of identified candidates is necessary to identify those with critical function in blastema formation.

## Materials and Methods

### Animal work

To reduce suffering as much as possible, the animals were placed in bath with anesthetic (0.03% benzocaine) until no response to tactile stimuli were observed, but strong heart rate persisted, prior to surgery. To promote as efficient and painless healing as possible, after limb amputation, the bone and skin tissue were trimmed to create a neat wound surface that would not undergo further tearing afterwards. Animals after limb amputation were placed in clean water, and monitored until waking, and in the next 24 hours for normal feeding and motility. Animals were kept in a continuous flow aquaria system, that was monitored daily for temperature, nitrogen levels, and pH.

The axolotl animal work was performed under permission granted in animal license number 24-9168.11-9/2009-3 conferred by the Animal Welfare Commission of the State of Saxony (Landesdirektion, Sachsen).

### Assembly and annotation of contigs

Quality control of EST-sequences was done using Phred [70] and remaining vector sequences were removed using the program Cross-match from the Phrap package. Quality controlled EST-sequences were assembled using TIGR Assembler [71]. EST-sequences from the two different laboratories (Randall Voss' and our lab), as well as from *Ambystoma mexicanum* and *Ambystoma tigrinum* were assembled individually. Annotation of hits was done using BLAST [72] to identify potential orthologs and using InterProScan [73] to find conserved domains and to extract Panther [74] Ontologies and Pathways for contig sequences. Contigs were named according to their closest homolog provided the E-value cut-off of 10^−3^ was met. Next to contig sequences from Randal Voss' and our lab, we included all axolotl sequences from the NCBI database, as well as fully sequenced clones from our lab for annotation, storage and array spotting. All sequences and accompanying information are stored in a MySQL database (the Axologle database accessible at http://est.age.mpg.de/). The interface, as well as all images and plots are scripted using Python. All new EST sequences have been deposited in GenBank. The accession numbers are available in the Table S5.

### Salamander array design

4x44 K arrays with the 60-mer oligonucleotides (probes) were produced by Agilent Technologies. Total of 43736 custom designed probes were targeted against 17452 assembled contigs of salamander sequences. For a subset of contigs it was not known which DNA strand is the coding strand. Including both strands of those contigs, in total 22753 different targets were probed with 1–2 probes per target (exceptionally up to 10). The 44 k microarray platform is accessible at GEO with the accession ID: GPL15341.

### Injury and tissue collection

Time-course after limb injury includes time points: 0, 3, 6, 9, 12, 24, 36, 52, 72, 120, 168, 288 and 528 hours after limb amputation (amp) and posterior lateral wound (lw). The corresponding amp and lw time points consist of contralateral limbs of the same animal. Use of left or right arm for the amp and lw injury was randomized. Each sample is a pool from 4–5 limbs. Replicates are biologically and technically independent. Animals for separate replicates come from separate clutches of eggs, but all the samples within replicates come from the same clutch. Each replicate was processed (samples collected, RNA purified, labeled and hybridized) individually.

For both, amputation and lateral injury samples the first cut was done at ¼ stylopod length behind the wrist using animals at the size 40–50 mm from nose to cloaca. In amputated stumps protruding bones were trimmed (≈ 0.5 mm). For regenerating samples, all tissue between 2 mm behind the amputation plane and the tip of the blastema was collected. For lateral wound samples an incision was made using scalpel through the posterior half of the lower arm such that ulna was cut through, but radius was not. No tissue was removed. At the appropriate time a 2 mm thick sliver of tissue immediately proximal to the cut was collected. Collected tissue was frozen immediately in liquid nitrogen, and kept at −80°C until further processing.

Limb bud samples were collected at mid-bud (the earliest stage when it was technically possible) and notch stage from 1.5–1.7 cm long animals 6 and 12 days after hatching, respectively. 40–50 buds were collected per sample.

### RNA processing and microarray hybridization

Total RNA was purified using Qiagen RNeasy midi kit. RNA was then DNase-treated and then re-purified using Qiagen RNeasy mini kit. RNA quality was confirmed using Agilent Bioanalyzer – the RIN values were at least 9.4.

Probes were labeled using Agilent Quick Amp Labeling Kit starting with 300 ng of total RNA. Procedure includes cDNA synthesis followed by T7 RNA-polymerase mediated linear amplification with simultaneous Cy3 dye incorporation.

After hybridization signal intensities were extracted using Agilent Technologies DNA microarray scanner and Feature-extraction software with 1-Color Gene Expression protocol. Data was transformed through background-correction using background detrending and multiplicative-detrending. All microarray data were deposited in the GEO-database and comply with MIAME standards (accession number GSE36451).

### Analysis of amputation versus lateral injury time course

Condition tree was created by hierarchical clustering of the averaged replicates using Pearson's correlation as the similarity measure and average-linkage as the clustering algorithm starting from the list of 39174 probes that in at least 6 samples had raw signal values above background (above 50).

#### A. Two-way ANOVA Analysis

Data were quantile normalized using GeneSpring 7.3. The two-way ANOVA over time course (between 3 and 528 hours, 12 time points and 3 replicates) and between the amputation and wound scenario resulted in 3645 probes with significant interaction effect (5% FDR). 600 of those shown none or only marginal differences in the wound time course (ANOVA p>0.05). A general characterization of the amputation time course profiles of these 600 probes (median expression over replicates) where performed using hierarchical clustering applying normalization of probe sets to a mean of zero and a standard deviation of one before Euclidean average-linkage clustering (Figure 4). GQLCluster [75], a time course-specific clustering package, where used to differentiate specific profiles. Here, the incipient cluster estimation using 4 stages and up to 8 clusters resulted in 5 clusters which have been analyzed using mixture-estimation and 10 iterations.

The profiles of the five resulting clusters (Figure 4) are divided in two clusters of continuous (cluster 2) and sudden (cluster 1) down-regulation, one cluster with heterogeneous short transient patterns (cluster 3), one cluster with transient up-regulation (cluster 4), and one with persistently up-regulated genes (cluster 5).

246 probes from clusters 3-5 were further characterized using LSD test p-values between consecutive time points in the time segment 3 hours to 72 hours of both amputation and wound time course. –log_10_ p-values were normalized to a mean of zero and a standard deviation of one and K-Mean clustered using a Euclidean distance function and K = 11 after Davies Bouldin K-estimation [76].

#### B. Pairwise comparison to identify additional regeneration-enriched gene set

Data normalization where performed in two steps. Values below 5.0 were set to 5.0. Afterwards the signal intensities where normalized by a percentile-shift (each measurement was divided by the 75th percentile of all measurements) and probes marginally expressed in >80% datasets where rejected (native signal value <50). Of the remaining 39173 probes 19017 changed in the amputation dataset (1-way ANOVA, 5% FDR), and 583 of these were differentially expressed between amputated and lateral-injury samples at three consecutive time points (2 fold and t-test p = 0.05).

Hierarchical average-linkage clustering matrix taking the mean over replicates, based on 39174 expressed probes, were performed using Pearson's correlation as the similarity matrix. Analysis was done using GeneSpring 7.3.

### qPCR

cDNA was made from 300 ng total RNA using SuperScript III polymerase. RNA samples corresponded to the three replicates that were used for the microarray. qPCR was performed using AB Applied Biosystems Power SYBR Green reaction mix in the final volume of 10 μl with the final primer concentration of 300 nm on the Lightcycler 480, Roche.

Primers used for PCR:

*DK35*: TCAGCCTATCCCAGACCTCTCAAG & GCTCTCCAGTTTCCACATTCCAAC

*DK45*: AACACTGTGGTGGTGGCTGTAAG &TGTAGTTATCTGGGGTCATCCTCAG

*Fgf10*: CACGGACGAATAACGGAGTTTCTG & CACGGACGAATAACGGAGTTTCTG

*Psca*: GCGAAGATGAAGGCTATCCTGGTC & TTGGTGGTTTGCTGGCAGTTG

*Rpl4*: TGAAGAACTTGAGGGTCATGG & CTTGGCGTCTGCAGATTTTTT

*Tgm5*: TTCGGGGTCTTGGGTGGTAAAG & TCCTGGGCACTGACAGCAATAG

*Wnt5a*: ATCCGACCAGCCTAAGCACACTTC & GCAGCACAGCAACAAAAGGAGC

*Wnt5b*: AGACAGTCGCTGACACCACAAGTG & TAACTCACAGATGGACCTGGGAGC

### *In situ* hybridization

*In situ* hybridization was carried out according to [77] with some modifications (detailed protocol at http://est.age.mpg.de). Limbs were collected at appropriate time after injury at the shoulder level. Tissues were fixed in MEMFA (0.1 M MOPS pH 7.4, 2 mM EGTA, 1 mM Mg SO_4_×7H_2_O and 3.7% formaldehyde) overnight at 4°C, washed in PBS, dehydrated by increasing ethanol series, Xylene and embedded into paraffin. 10 µm sections were de-waxed, rehydrated and hybridized with 0.5 and 2 kb long *in vitro* transcribed DIG – labeled probes in hybridization buffer (50% formamide, 10% dextran, 5× SSC, 0.1% Tween, 1 mg/ml yeast RNA, 100 g/ml heparin, 1× Denhardt's, 0.1% CHAPS, 5 mM EDTA) at a concentration of 100–600 ng/ml for at least 16 hours at 70°C. Slides were washed 3× for 1 hour each and overnight at 70°C in post-hybridization buffer (50% formamide, 5× SSC, 0.1% Tween) followed by wash buffer (50% formamide, 2× SSC, 0.1% Tween) twice for 1 hour each. The signal was detected with anti-DIG Fab fragments conjugated with alkaline phosphatase (Roche) and BM purple (Roche).

The complete detailed protocol is accessible from the *in situ* database at http://est.age.mpg.de.

## Supporting Information

Figure S1 **Quality assessment of the microarray expression profiles.** A. Correlation coefficients between 8151 probe-pairs targeting the same contig. Correlation coefficients were calculated for those probe-pairs that showed statistically significant expression changes (ANOVA at 5% FDR) during the time course. B. Gene tree of the probes for a subset of cell cycle genes represented on the array created by hierarchical clustering using Pearson's correlation as the similarity measure and average-linkage as the clustering algorithm. In general, up-regulation of genes with a function in G1-phase of the cell cycle precedes the G2/M-related genes by approximately 2 days. Amputation samples often show a late phase of expression that is not evident in the lateral wound samples.
(PDF)

Figure S2 **STRING analysis of the two-way ANOVA gene set showing functional associations including the low confidence interactions (score ≥0.15).** Red and yellow balls are associated with response to oxidative and cellular stress. Blue balls denote chromatin-modifying genes. Dark green balls denote the epithelial cluster. Pink balls denote the limb development cluster. Red frames mark genes of the 10^th^ p-value cluster. Thicker lines represent higher confidence interactions. Large balls represent gene families where a protein structure is available.
(PDF)

Figure S3 ***In situ*** **hybridization on the longitudinal sections of limbs with lateral wound contralateral to the amputated samples in** **Figure 6** **.** A. Genes up-regulated in the wound epidermis of amputated limbs (See Figure 6A) show modest or no up-regulation in the lateral wound. B. Genes up-regulated in the mesenchyme of amputated limbs (See Figure 6B) show modest or no up-regulation in the lateral wound. *Hnrnpl* and *DK40* show limited up-regulation in the lateral wound while other genes remain at the basal level. Arrows point to the injured place. Posterior side of the limb is at the bottom of the pictures, distal is to the right. C. Expression of *Wnt5a* in the lateral wound is not detectable by *in situ* hybridization. D. Expression of *DK45* in the lateral wound is not detectable by *in situ* hybridization.
(PDF)

Figure S4 **Microarray expression profiles of pluripotency factors** **and epigenetic regulators.** A. Expression profiles of *Oct4, Sox2, Nanog, Klf4* and *c-Myc*. B. Expression profiles of chromatin modifiers: *Smarcd1, Smarcd4, Hmgb3* and *Hmgb2*. Each line represents the trace for an individual probe for the gene averaged over three replicates with the error bars representing standard deviation. Colors of lines differ only to distinguish between traces.
(PDF)

Figure S5 **STRING analysis showing functional associations between regeneration-enriched genes selected by pairwise comparison.** All interactions including the low confidence ones (score ≥0.15) are shown. ECM remodeling network including MMPs and elastase is shown with yellow balls, epithelial organization network in brown and the limb bud network including WNT5A, MSX1, LHX2 in orange color balls. Thicker lines represent higher confidence interactions. Large balls represent gene families where a protein structure is available.
(PDF)

Figure S6 **Heat map of 3 clusters obtained by K-means clustering of 395 probes that were selected either by two-way ANOVA or by pairwise comparison relating gene expression after injury with the expression in the limb bud.** Gene trees were made on each of the clusters separately using Pearson's correlation as similarity measure. Up-regulation is indicated by red, down-regulation by green color. A. Cluster 1 – genes expressed at low levels in the limb bud, and are up-regulated by lateral injury. B. Cluster 2 – genes expressed at low levels in the limb bud, and are amputation-specific. C. Cluster 3 – genes highly expressed in the limb bud and amputation specific.
(PDF)

Table S1 **List of 246 probes representing amputation-specific genes that were selected by two-way ANOVA analysis.** Probe name: name of the oligo-nucleotide spotted on the microarray (sequence accessible at GEO), Target name: name of he target where the prefixes “a.” and “s.” distinguish between different strands of the contig, Contig name: name of the contig (sequence accessible at http://est.age.mpg.de), Cluster (expression) K = 5: Clusters depicted in Figure 4, Cluster (-log10 p-value) K = 11: Clusters depicted in Figure 7, Cluster (limb bud expression): Clusters depicted in Figure 9, Correlation coefficient: correlation coefficient between the probes for the same target. Probes highlighted in italicized print target the antisense-strand of the annotated gene.
(XLS)

Table S2 **List of 173 probes representing amputation-enriched genes that were selected by the pairwise comparison.** Probe name: name of the oligo-nucleotide spotted on the microarray (sequence accessible at GEO), Target name: name of the target where the prefixes “a.” and “s.” distinguish between different strands of the contig, Contig name: name of the contig (sequence accessible at http://est.age.mpg.de), Cluster (limb bud expression): Clusters depicted in Figure 9, Correlation coefficient: correlation coefficient between the probes for the same target. Probes highlighted in italicized print target the antisense-strand of the annotated gene.
(XLS)

Table S3 **Gene ontology enrichment analysis on the list of 56 amputation-specific/enriched genes with a low expression in the limb bud.** Enrichment was calculated using g:Profiler on the list of human RefSeq protein IDs form the limb bud expression clusters 1–3. 27 RefSeq IDs from cluster 1, 20 IDs from cluster 2 and 64 IDs from cluster 3 were considered to calculate the enrichment. Columns represent: P-value: enrichment P-value, #Array: total number of genes on the array associated to functional term, #Query: total number of genes in the input list, #List: number of genes in the input list that are associated to functional term, GO ID: GO term identifier, Type: type of functional evidence (BP  =  biological process, CC  =  cell compartment, MF  =  molecular function, bi: BioGRID interaction data, hp: human phenotype ontology, ke: KEGG pathways), GO term: term name (the vertical alignment of the term name depicts its depth in the GO term hierarchy), Gene list: list of genes present in the cluster that are associated with the particular GO term.
(XLS)

Table S4 **Comparison of the amputation-specific/-enriched genes with the published gene sets identified by comparative gene expression profiling of limb regeneration.** All gene sets were compared via the associated human homolog RefSeq IDs. Column D indicates genes that were also found in the list of 173 genes (corresponding to 371 probe sets) up-regulated at either 5 days or 14 days after injury in [8]. Column E indicates genes that were found in a list of 110 genes that were up-regulated in inervated relative to denervated limbs at either 5 days or 14 days after amputation in [8]. Column F indicates genes that were found in a list of 377 genes (corresponding to 571 probe sets) that were up-regulated in an amputated limb relative to the uninjured limb and also relative to a flank skin injury [10]. Column G indicates genes that were found in the list of 58 genes (corresponding to 195 probes) that were up-regulated in a regenerative epidermis relative to a non-regenerative epidermis [9].
(XLS)

Table S5 **The list of GenBank accession numbers and EST identifiers for new ESTs used in this study that were not submitted previously.** dbEST ID: identifier of a dbEST database entry, EST name: name of the EST sequence submitted to the GenBank, GenBank accession: accession number of a GenBank sequence database entry.
(XLS)
