# Supplementary figures and images for: Comparative Transcriptional Profiling of the Axolotl Limb Identifies a Tripartite Regeneration-Specific Gene Program
Source: PLoS One. 2013 May 1;8(5):e61352. doi: 10.1371/journal.pone.0061352 (PMC3641036; doi:10.1371/journal.pone.0061352)

Figure S1

A

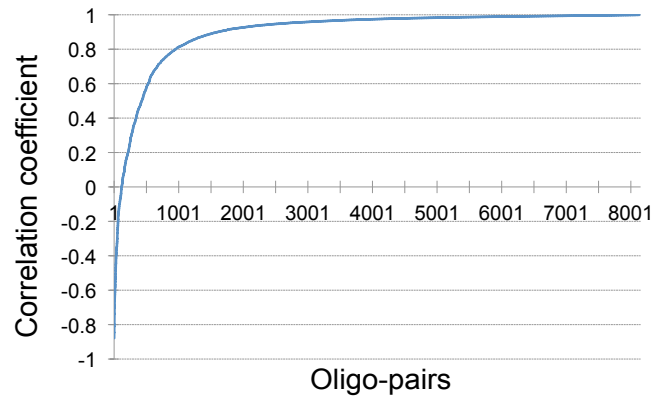

B

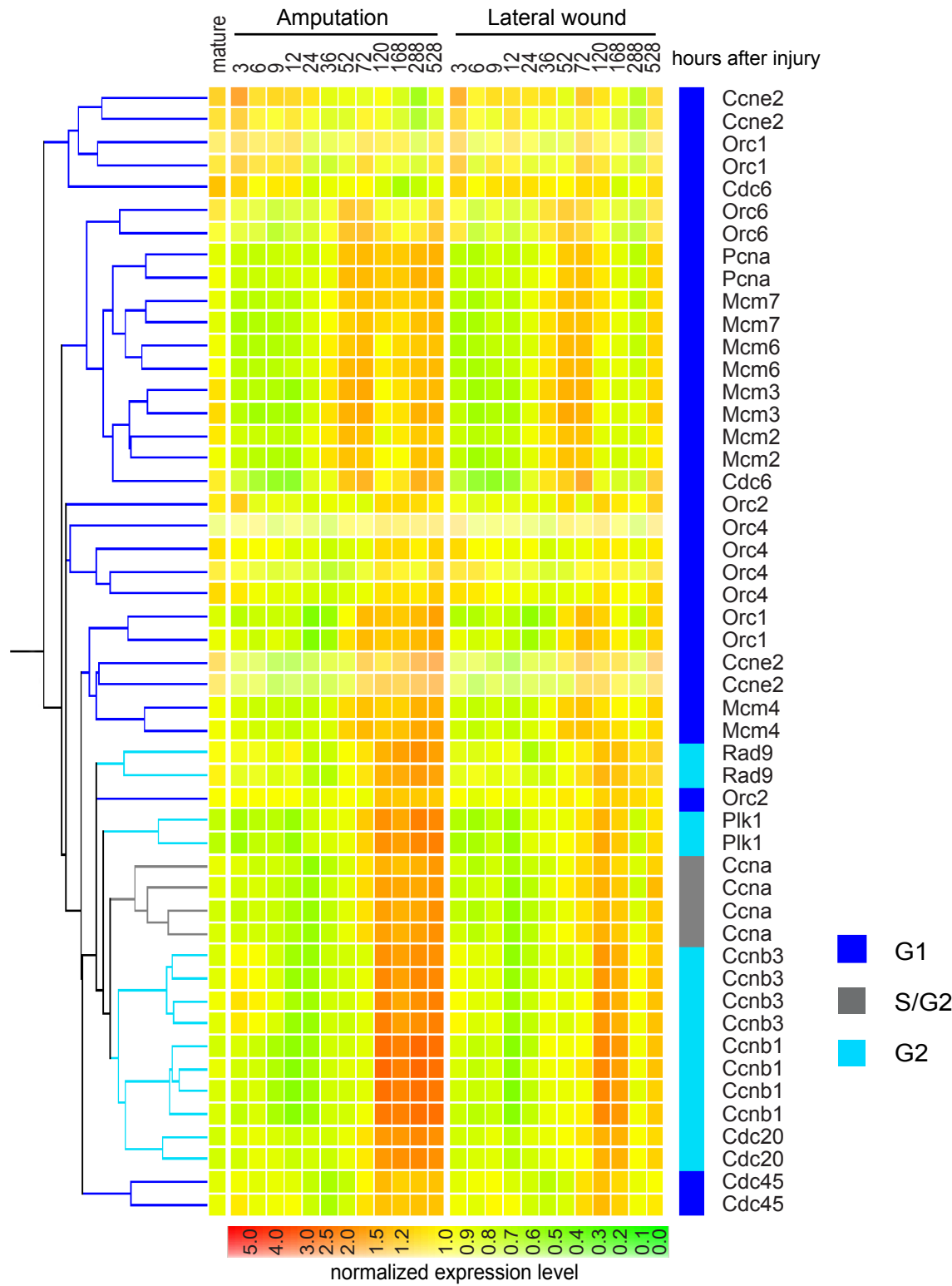

Supplement: Figure S1 — Quality assessment of the microarray expression profiles. A. Correlation coefficients between 8151 probe-pairs targeting the same contig. Correlation coefficients were calculated for those probe-pairs that showed statistically significant expression changes (ANOVA at 5% FDR) during the time course. B. Gene tree of the probes for a subset of cell cycle genes represented on the array created by hierarchical clustering using Pearson's correlation as the similarity measure and average-linkage as the clustering algorithm. In general, up-regulation of genes with a function in G1-phase of the cell cycle precedes the G2/M-related genes by approximately 2 days. Amputation samples often show a late phase of expression that is not evident in the lateral wound samples. (PDF) [file pone.0061352.s001.pdf]

Figure S2

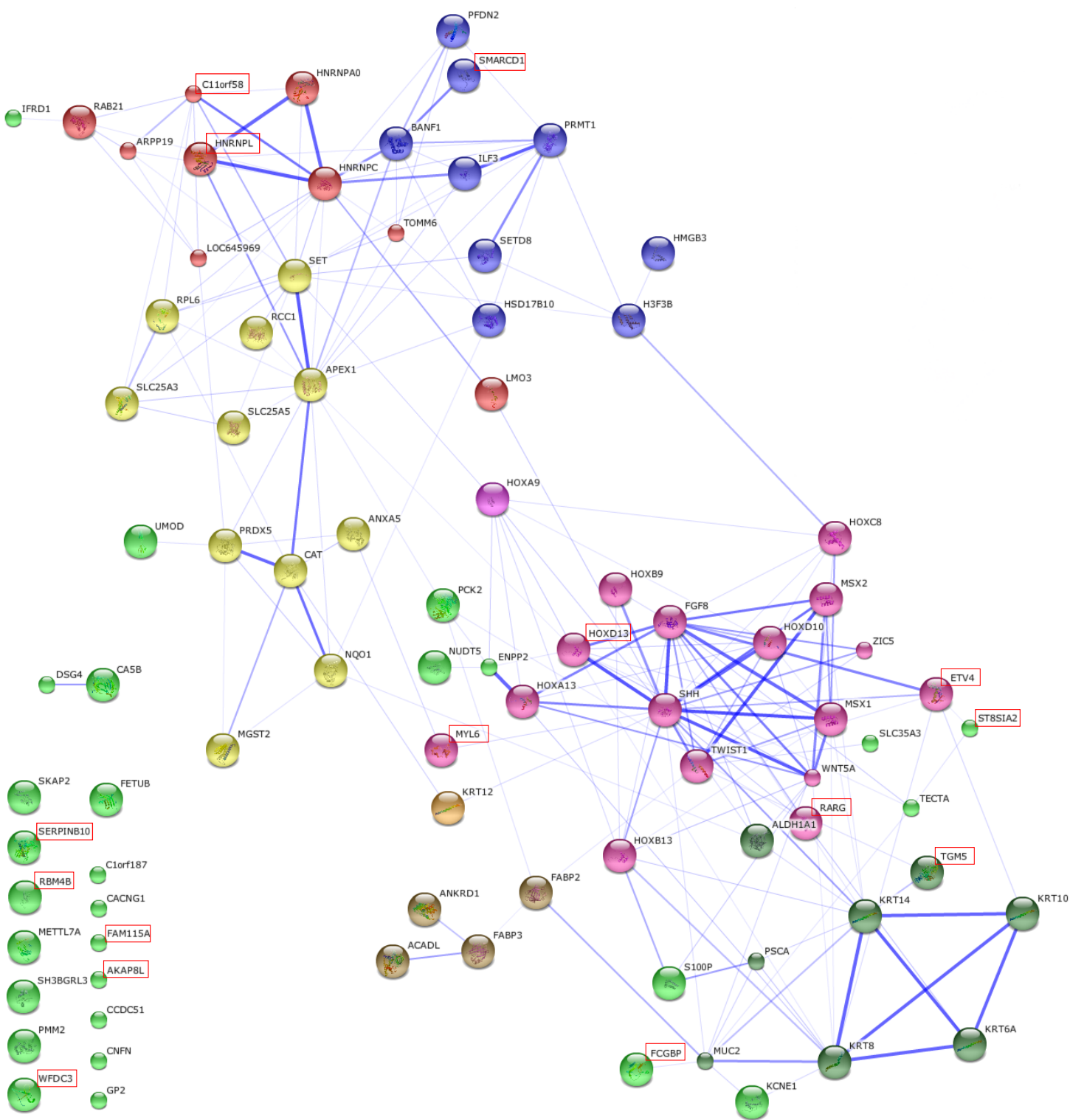

Supplement: Figure S2 — STRING analysis of the two-way ANOVA gene set showing functional associations including the low confidence interactions (score ≥0.15). Red and yellow balls are associated with response to oxidative and cellular stress. Blue balls denote chromatin-modifying genes. Dark green balls denote the epithelial cluster. Pink balls denote the limb development cluster. Red frames mark genes of the 10th p-value cluster. Thicker lines represent higher confidence interactions. Large balls represent gene families where a protein structure is available. (PDF) [file pone.0061352.s002.pdf]

Figure S3

A

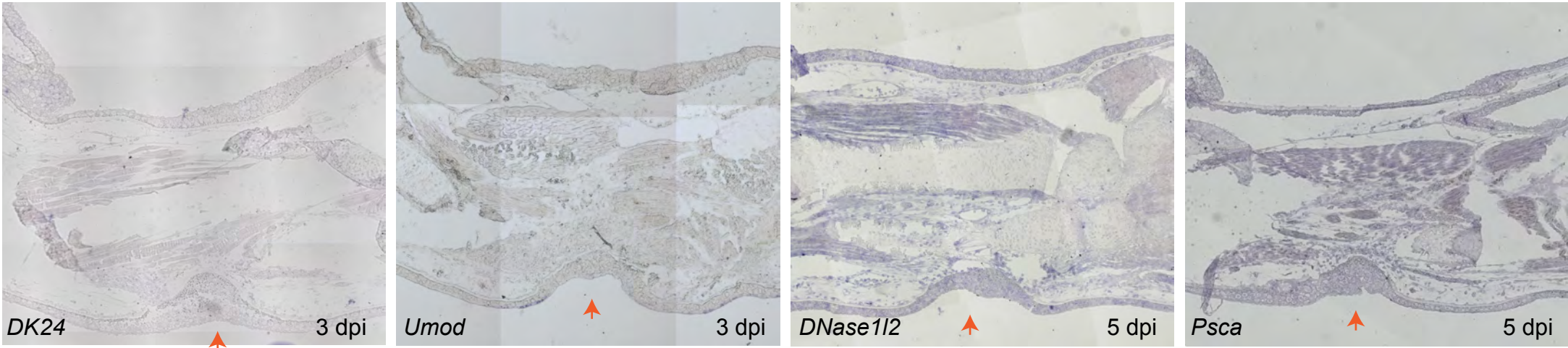

B

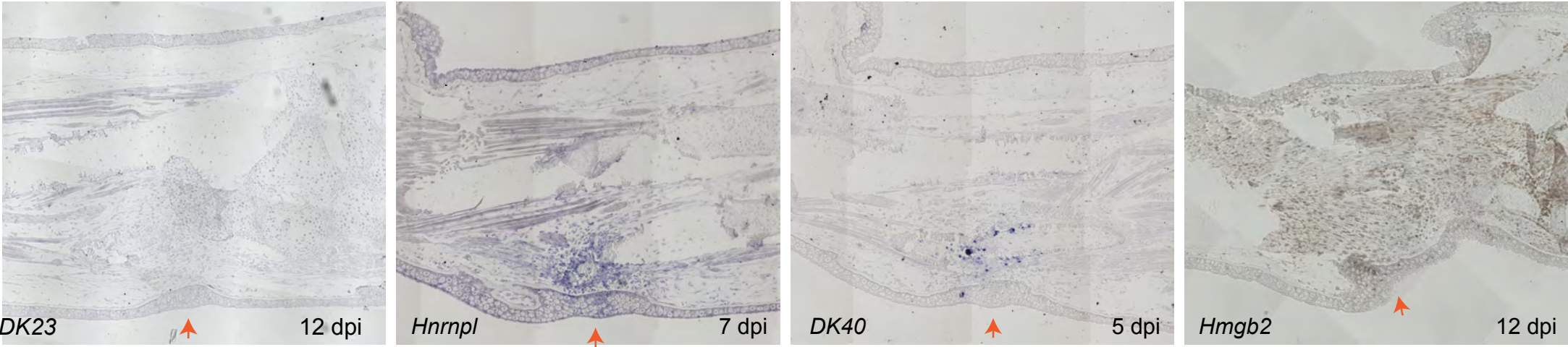

C

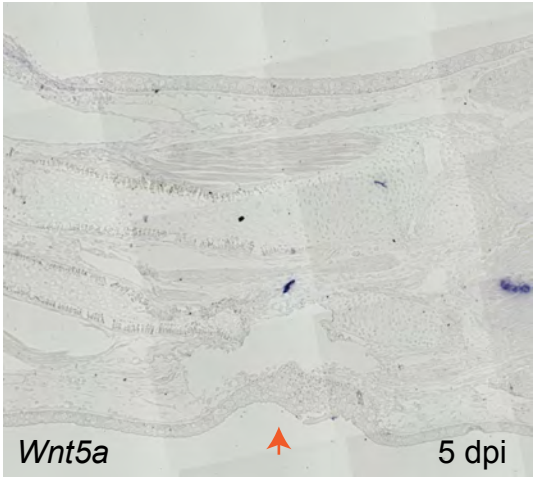

D

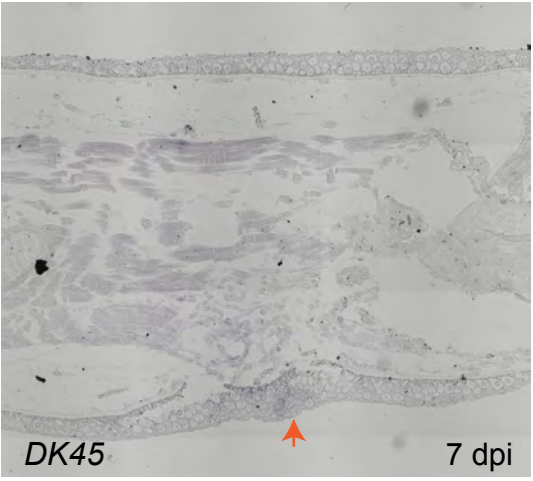

500  $\mu$ m

Supplement: Figure S3 — In situ hybridization on the longitudinal sections of limbs with lateral wound contralateral to the amputated samples in Figure 6 . A. Genes up-regulated in the wound epidermis of amputated limbs (See Figure 6A) show modest or no up-regulation in the lateral wound. B. Genes up-regulated in the mesenchyme of amputated limbs (See Figure 6B) show modest or no up-regulation in the lateral wound. Hnrnpl and DK40 show limited up-regulation in the lateral wound while other genes remain at the basal level. Arrows point to the injured place. Posterior side of the limb is at the bottom of the pictures, distal is to the right. C. Expression of Wnt5a in the lateral wound is not detectable by in situ hybridization. D. Expression of DK45 in the lateral wound is not detectable by in situ hybridization. (PDF) [file pone.0061352.s003.pdf]

Figure S4

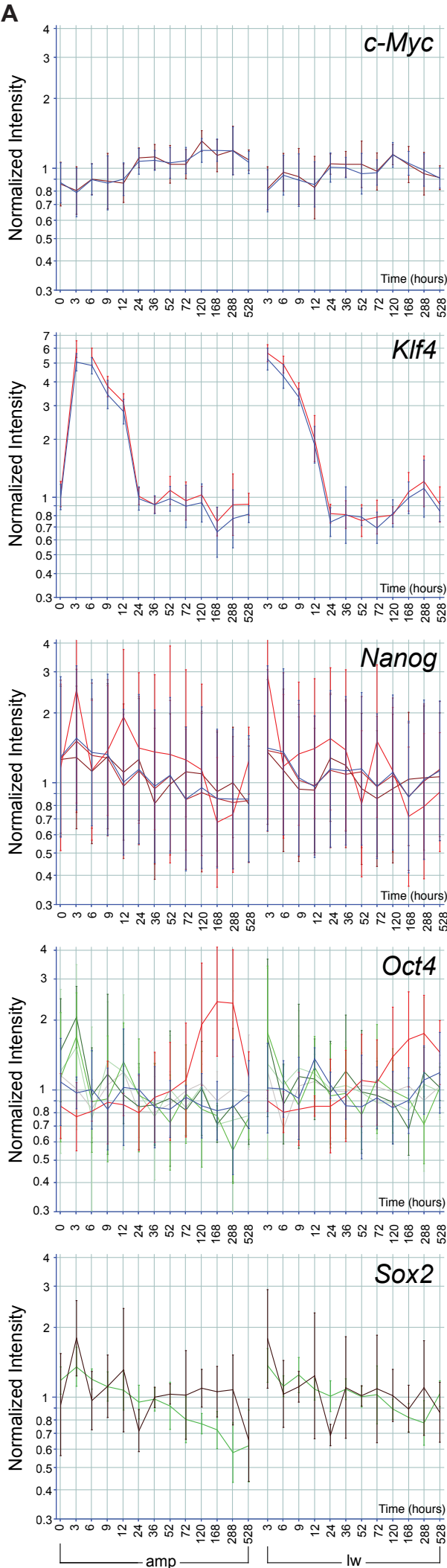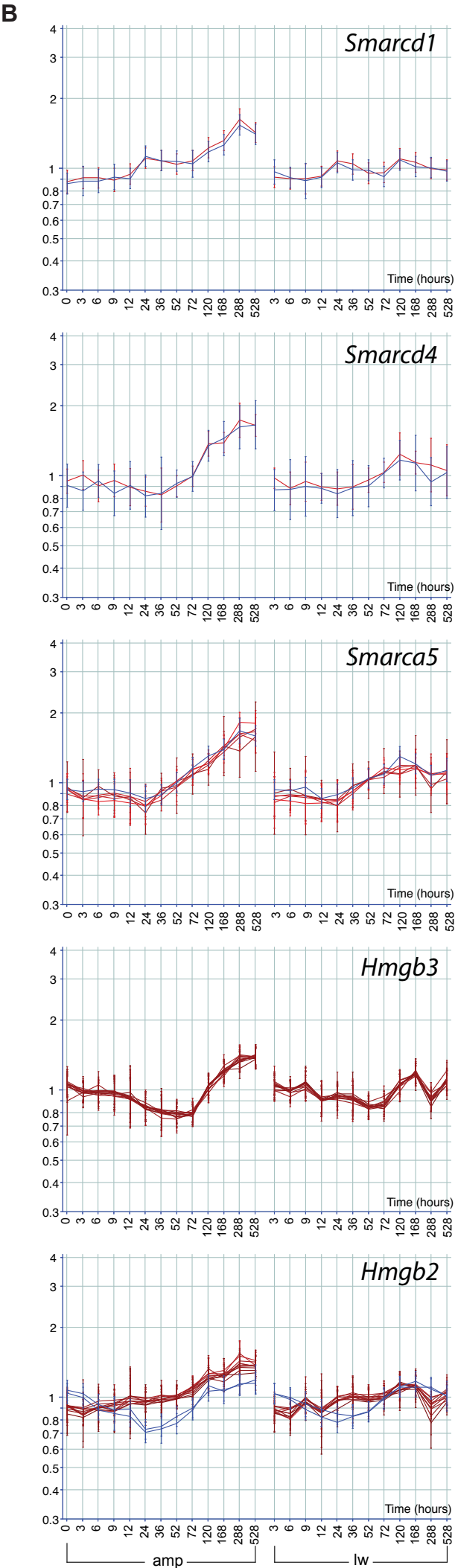

Supplement: Figure S4 — Microarray expression profiles of pluripotency factors and epigenetic regulators. A. Expression profiles of Oct4, Sox2, Nanog, Klf4 and c-Myc. B. Expression profiles of chromatin modifiers: Smarcd1, Smarcd4, Hmgb3 and Hmgb2. Each line represents the trace for an individual probe for the gene averaged over three replicates with the error bars representing standard deviation. Colors of lines differ only to distinguish between traces. (PDF) [file pone.0061352.s004.pdf]

Figure S5

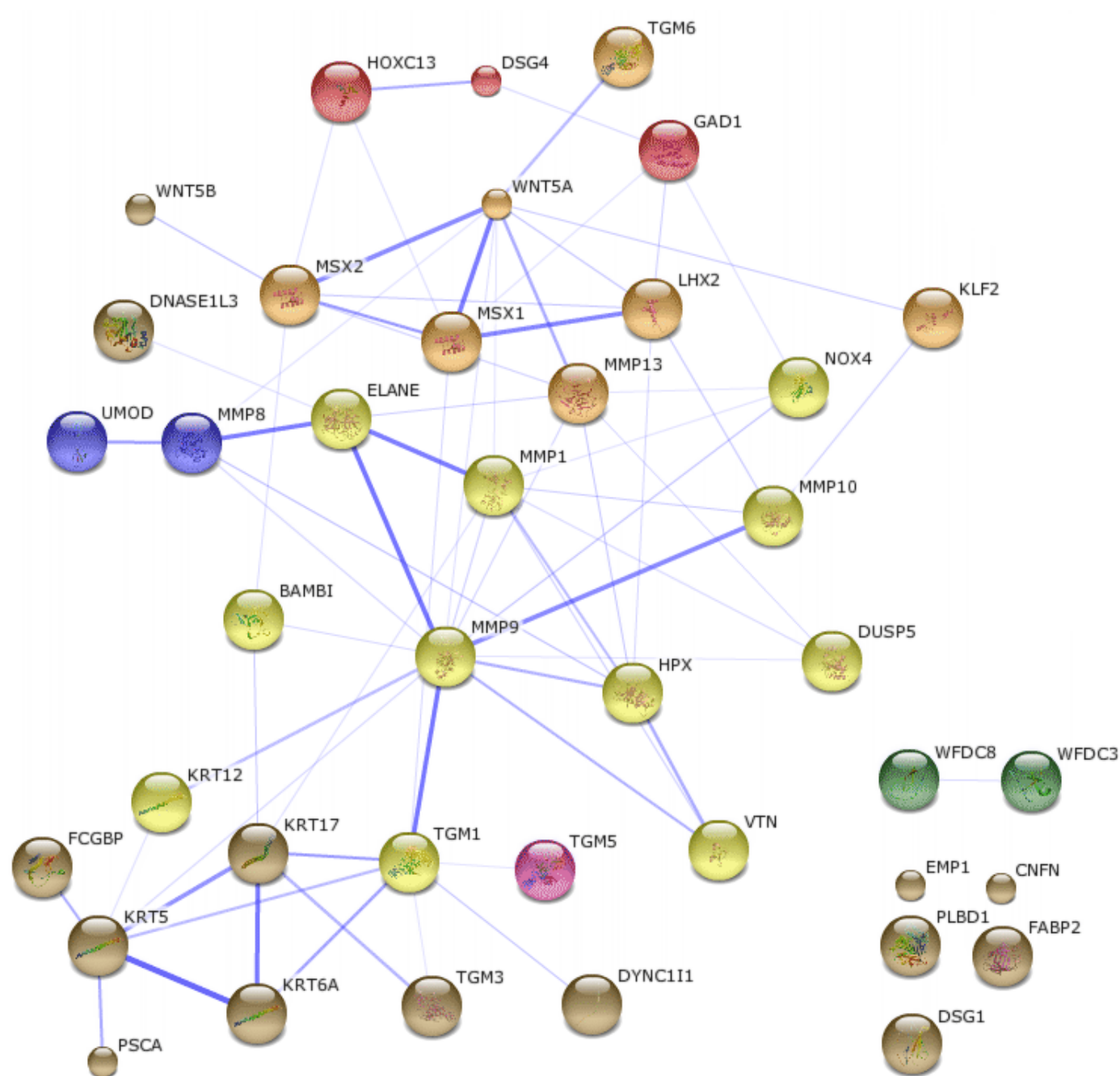

Supplement: Figure S5 — STRING analysis showing functional associations between regeneration-enriched genes selected by pairwise comparison. All interactions including the low confidence ones (score ≥0.15) are shown. ECM remodeling network including MMPs and elastase is shown with yellow balls, epithelial organization network in brown and the limb bud network including WNT5A, MSX1, LHX2 in orange color balls. Thicker lines represent higher confidence interactions. Large balls represent gene families where a protein structure is available. (PDF) [file pone.0061352.s005.pdf]

### Figure S6

A

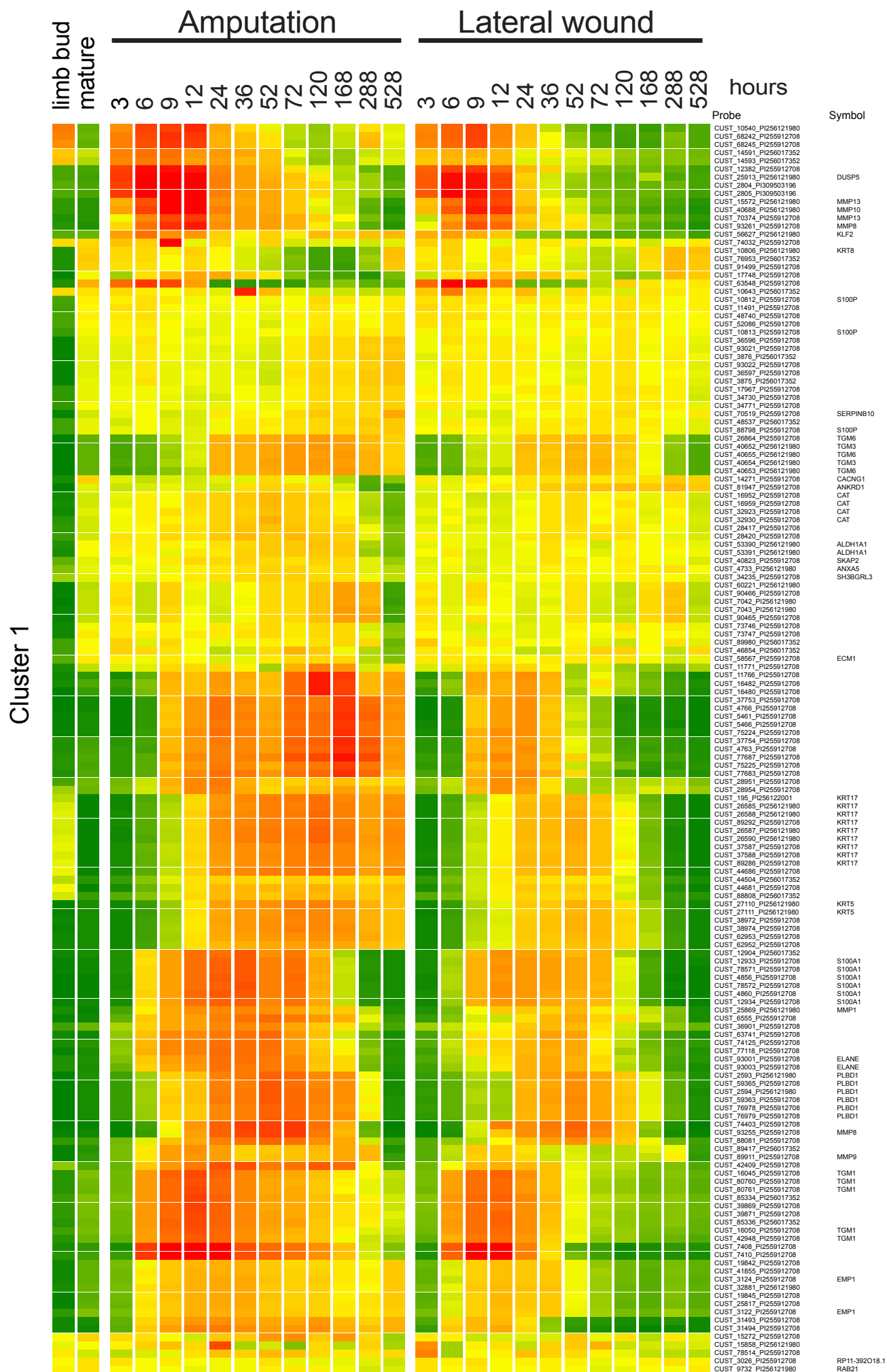

B

Cluster 2

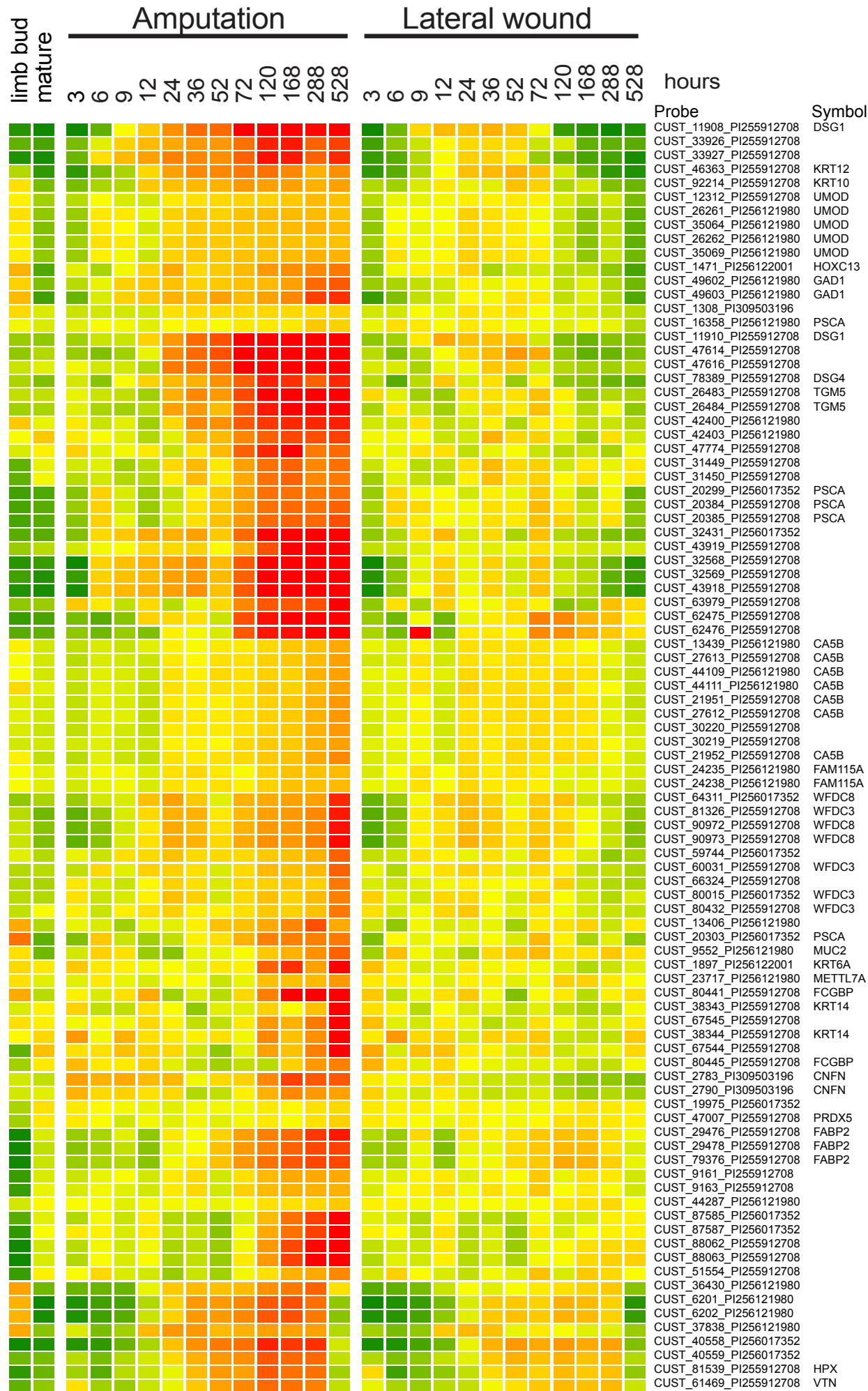

C

Cluster 3

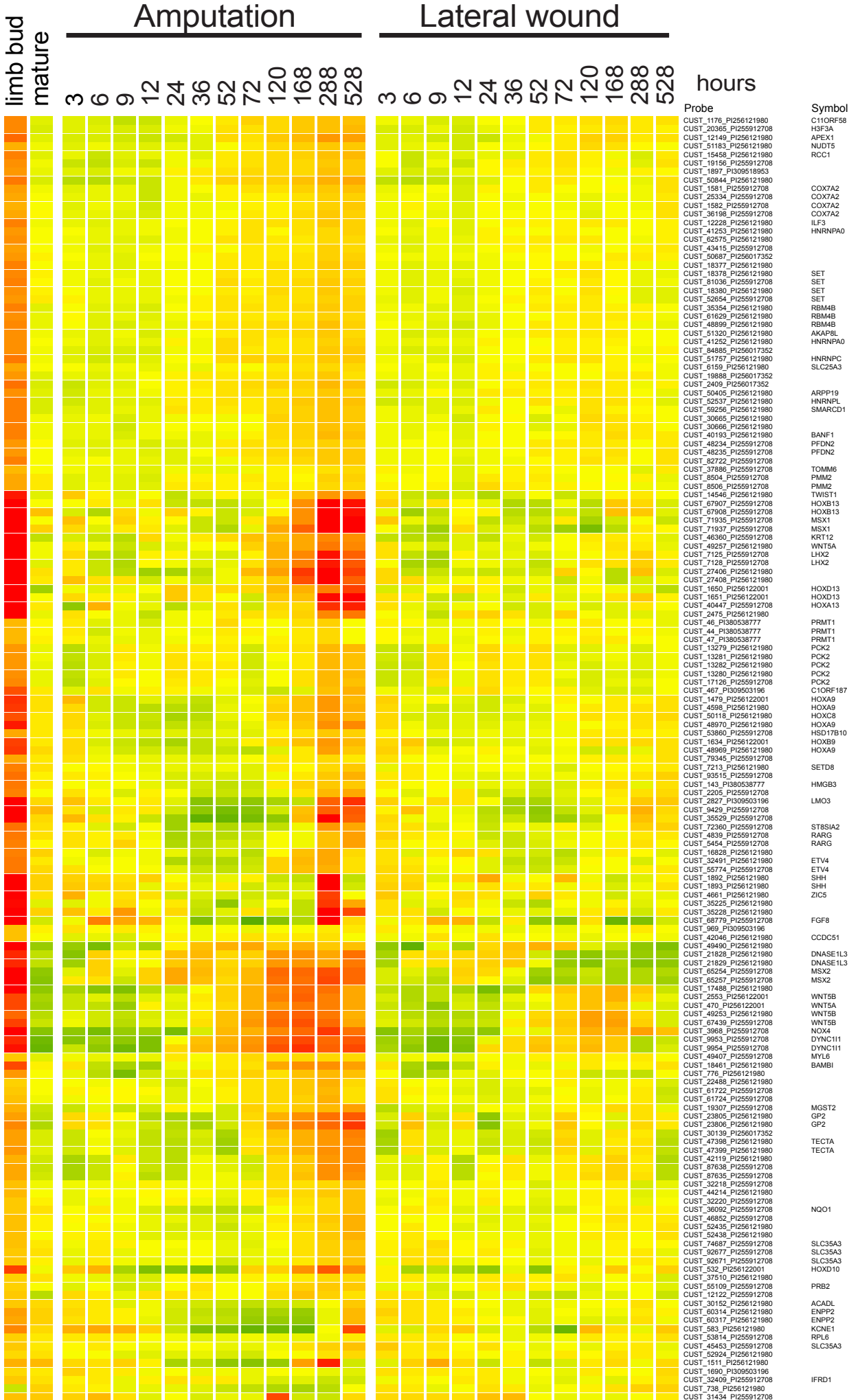

Supplement: Figure S6 — Heat map of 3 clusters obtained by K-means clustering of 395 probes that were selected either by two-way ANOVA or by pairwise comparison relating gene expression after injury with the expression in the limb bud. Gene trees were made on each of the clusters separately using Pearson's correlation as similarity measure. Up-regulation is indicated by red, down-regulation by green color. A. Cluster 1 – genes expressed at low levels in the limb bud, and are up-regulated by lateral injury. B. Cluster 2 – genes expressed at low levels in the limb bud, and are amputation-specific. C. Cluster 3 – genes highly expressed in the limb bud and amputation specific. (PDF) [file pone.0061352.s006.pdf]
